# MSH1-induced heritable enhanced growth vigor through grafting is associated with the RdDM pathway in plants

**DOI:** 10.1038/s41467-020-19140-x

**Published:** 2020-10-22

**Authors:** Hardik Kundariya, Xiaodong Yang, Kyla Morton, Robersy Sanchez, Michael J. Axtell, Samuel F. Hutton, Michael Fromm, Sally A. Mackenzie

**Affiliations:** 1 Department of Agronomy and Horticulture, University of Nebraska, Lincoln, NE USA; 2 Department of Biology, The Pennsylvania State University, University Park, PA USA; 3 EpiCrop Technologies, Inc., Lincoln, NE USA; 4 Gulf Coast Research and Education Center, IFAS, University of Florida, Wimauma, FL USA; 5 Departments of Biology and Plant Science, The Pennsylvania State University, University Park, PA USA

**Keywords:** Field trials, Agricultural genetics, DNA methylation, Plant molecular biology

## Abstract

Plants transmit signals long distances, as evidenced in grafting experiments that create distinct rootstock-scion junctions. Noncoding small RNA is a signaling molecule that is graft transmissible, participating in RNA-directed DNA methylation; but the meiotic transmissibility of graft-mediated epigenetic changes remains unclear. Here, we exploit the *MSH1* system in *Arabidopsis* and tomato to introduce rootstock epigenetic variation to grafting experiments. Introducing mutations *dcl2*, *dcl3* and *dcl4* to the *msh1* rootstock disrupts siRNA production and reveals RdDM targets of methylation repatterning. Progeny from grafting experiments show enhanced growth vigor relative to controls. This heritable enhancement-through-grafting phenotype is RdDM-dependent, involving 1380 differentially methylated genes, many within auxin-related gene pathways. Growth vigor is associated with robust root growth of *msh1* graft progeny, a phenotype associated with auxin transport based on inhibitor assays. Large-scale field experiments show *msh1* grafting effects on tomato plant performance, heritable over five generations, demonstrating the agricultural potential of epigenetic variation.

## Abstract (web summary)

The meiotic transmissibility and progeny phenotypic influence of graft-mediated epigenetic changes remain unclear. Here, the authors use the *msh1* mutant in the rootstock to trigger heritable enhanced growth vigor in *Arabidopsis* and tomato, and show it is associated with the RNA-directed DNA methylation pathway.

## Introduction

Plants display remarkable adaptability to diverse and variable environments. This plasticity is evident in plant seed dispersal, e.g., where seeds or spores successfully relocate and establish at sites quite different from where they originate. Rapid environmental responsiveness in plants is thought to arise via epigenomic changes as a means of achieving phenotype plasticity within these sessile organisms^1^. Epigenetic chromatin variation can encompass small RNA (sRNA) expression changes, DNA methylation repatterning, posttranslational modification of histone proteins, and variant histone composition within nucleosomes. Although all of these forms of nongenetic variation have been observed in plants following environmental fluctuations^2^, the extent and means by which they coordinately effect programmed adjustment in gene networks is still not understood.

Cytosine methylation is a chromatin modification that influences gene expression and transposable element (TE) activity, with some degree of transgenerational inheritance^3,4^. Site-directed changes in DNA methylation within the genome are controlled, at least in part, by small interfering RNA (siRNA)-directed DNA methylation (RdDM) processes^5^. Plant noncoding RNAs direct de novo cytosine methylation in any sequence context (CG, CHG, CHH) by the methyltransferase DRM2. Once targeted cytosines undergo methylation, other methyltransferase enzymes can maintain DNA methylation patterns in association with subsequent rounds of DNA replication. This reinforcing CG methylation patterning is maintained by MET1, whereas CHG and CHH methylation is maintained variably by CMT2, CMT3, and DDM1^6,7^. These methylation patterns are important determinants of local histone modification behavior, thus serving to integrate components of local chromatin architecture.

Recent research has provided important details of sRNA mobility within the plant, with long distance transmission mediated through vascular tissues.^8^ Epigenetic effects directed by siRNA action can be detected by implementing grafting studies. Studies by Molnar et al.^9^ first showed in *Arabidopsis* that scion to root transmission of siRNA could direct methylation changes at identified TE sites. Another study demonstrated that scion-originating siRNAs can influence methylation changes at thousands of root loci^10^. However, these RdDM-mediated changes were not associated with gene expression effects. The majority of previous graft studies of epigenetic phenomena, in *Arabidopsis* or other plant species, have focused on effects within the rootstock or scion, but not heritably to graft progeny. A recent study of epigenomic response to grafting in *Brassica* investigated epigenetic effects transmitted to scion clonal propagants, but did not include reproductive progeny in the study^11^. Similarly, inter-specific grafting of potato was shown to cause changes in the tuber that could be vegetatively propagated, but without sexual transmission^12^. Whether siRNA-mediated changes are associated with gene expression and are directly heritable through meiosis are the subject of continued debate.

The *MSH1* system provides a means to trigger epigenetic reprogramming in the plant. *MSH1* is a plant-specific gene that encodes a mitochondrial- and plastid-targeted protein^13^. Disruption of MSH1 function within the plastid leads to variation in plant growth rate, flowering time, response to short day length, leaf morphology, variegation, and stress response, phenotypes that are reproducibly observed across a range of plant species^14,15^. The *msh1* mutant state is dependent on *HISTONE DEACETYLASE 6* (*HDA6*) and the methyltransferase *MET1*, and results in genome-wide DNA methylation repatterning, changes in siRNA expression, and heritable nongenetic memory^16^. Graft experiments incorporating the *msh1* mutant as rootstock give rise to progeny that display enhanced growth vigor and seed yield as a heritable phenotype^17,18^. The possible relationship of this enhanced vigor to epigenetic processes was the focus of these investigations.

Here we present comparative analyses of the *msh1* graft phenomenon in *Arabidopsis* and tomato, two species in which successful grafting is feasible and *msh1* phenotypes are established^17,18^. We show that the enhanced plant vigor phenotypes from *msh1* grafting experiments can be heritably reproduced at field scale. The graft effects are dependent on siRNA transmission from the *msh1* rootstock, driving targeted cytosine methylation repatterning in graft progeny. Incorporation of siRNA-null mutants to the *msh1* rootstock obviates the vigor phenotype in graft progeny and delineates RdDM-targeted genes. Prominent gene network targets of methylation repatterning include auxin-related pathways, so that altered expression of auxin-response genes and increased lateral root growth contribute to the increased plant vigor phenotype. These data inform a feasible implementation of epigenomic strategies for agricultural improvement.

## Results

### Grafting to *msh1* leads to heritable and scalable enhanced growth response

**Fig. 1:**
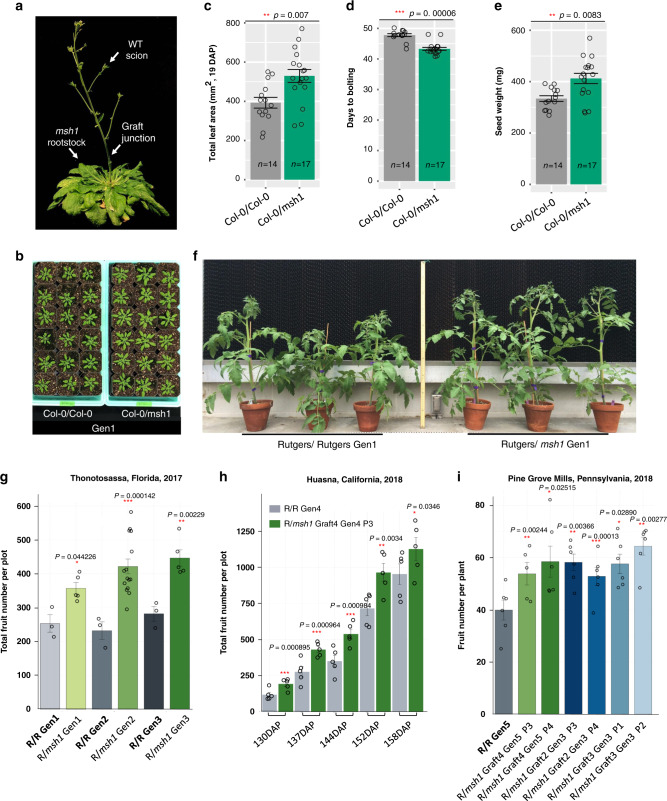
Grafting to *msh1* rootstock produces increased plant vigor that is heritable and scales to field production. **a** A grafted *Arabidopsis* plant, with graft junction indicated. **b** Phenotype of grafted progenies (first generation) from control graft Col-0/Col-0. The Col-0/*msh1* graft used Col-0 as scion and *msh1* as rootstock. Seedling stage photo at 27 days after planting (DAP). **c** Total leaf area (19 DAP), **d** days to bolting, and **e** seed weight (mg) in grafted progenies (first generation). **c**–**e** Bars represent means ± SE, *n* represents number of plants in each population. The Mann–Whitney *U*-test with two-sided alternative hypothesis was used to test significance of the difference of mean in each comparison. **f** Phenotype of tomato graft progenies (first generation) from control graft Rutgers/Rutgers and Rutgers/*msh1*, with Rutgers as scion and *MSH1-*RNAi as rootstock. The photograph shows 7-week-old plants. The yield and plant height of these plants was reported in Yang et al.^18^. **g** Total fruit number from tomato graft progenies (the first, second, and third generation) for Rutgers/Rutgers and Rutgers/*msh1* grafting events in the 2017 Florida field experiment. Figure shows the average total fruit number per plant per plot, with each plot represented by a single dot. Bars represent means ± SE (*n* = 3 for R/R control, *n* = 6 for R/*msh1* gen1, *n* = 15 for R/*msh1* gen2, and *n* = 6 for R/*msh1* gen3). **h** Cumulative total fruit number for tomato graft progenies (fourth generation) for Rutgers/Rutgers and Rutgers/*msh1* grafts in the 2018 California field experiment. Figure shows the average total fruit number per plot at 5 time points (130-158DAP), with the 12 plants measured each plot represented by a single dot, and bars represent means ± SE (*n* = 6). **i** Total fruit number per plant for tomato graft progenies (fifth generation, with a third-generation progeny from a different grafting event) for Rutgers/Rutgers and Rutgers/*msh1* grafts in the 2018 Pennsylvania field experiment. Figure shows the average total fruit number per plant, with each plant represented by one dot; bars represent means ± SE (*n* = 6). **g**–**i** Tests for significant differences in line means (each Rutgers/*msh1*group compared against its corresponding Rutgers/Rutgers control) were performed using linear mixed hypothesis, with the model *y*_*ij*_ ∼ genotype_*i*_ + block_*j*_ + *e*_*ij*_, where genotype_*i*_ is treated as a fixed effect and block_*j*_ is treated as a random effect and *e*_*ij*_ is the residual error, implemented by the lmer function in the R package lmerTest (version 3.1.1) Significance codes: *p < 0.05, **p < 0.01, ***p < 0.001. Source data underlying **c**–**e** and **g**–**i** are provided as a Source Data file.

*Arabidopsis* floral stem grafts between Col-0 wild type and *msh1* mutants can be generated with the mutant as rootstock and Col-0 as scion (designated Col-0/*msh1*) (Fig. 1a and Supplementary Fig. 1). For these experiments, Col-0/Col-0 grafts serve as control. Seed progenies from Col-0/*msh1* grafts (generation 1) show markedly enhanced vigor compared to control graft progeny, with plants displaying a larger rosette, early flowering, and increased total seed weight (Fig. 1b–e) as was reported previously^17^. This phenotype was heritable to the second generation after grafting (Supplementary Fig. 2a). Similar growth vigor has been observed in *msh1* grafts in tomato^18^. However, these observations were made from very limited progeny sizes under controlled environment conditions. A more robust test of this heritable enhancement-through-grafting (HEG) phenomenon involved graft experiments in tomato with multi-generation, multi-year, and multi-location field trials. These experiments used transgenic Rutgers MSH1-RNAi lines as rootstock and Rutgers wild type as scion (designated R/*msh1;* Supplementary Fig. 1). Seed progenies from R/*msh1* grafts (generation 1) were confirmed to display markedly enhanced growth vigor (Fig. 1f) in the greenhouse. We self-pollinated the *msh1* and control graft progenies to advance generations, and field trials were carried out in Florida (2017) with generations 1, 2, and 3. Significant increases in both fruit yield and fruit number were observed in the graft progenies, with one second-generation graft progeny line showing increases in fruit yield of 78% (average fruit yield 64.1 kg per plot) relative to its graft control (average fruit yield 36.0 kg per plot; Supplementary Fig. 3). This increase was heritable through self-crossing with no selection introduced (Fig. 1g).

The *msh1* graft process created variation in progeny in all three generations for fruit yield, fruit number and perhaps in biotic stress tolerance (Supplementary Fig. 3). We then evaluated performance of the fourth-generation graft progeny in a 2018 field trial in California. Fig. 1h and Supplementary Fig. 4 show results from a graft progeny line with significantly increased average total fruit yield and number per plot over 5 time points (from 130 to 158DAP), suggesting that the HEG effect occurs at an early stage and maintains through reproductive development. In a third field trial, conducted in Pennsylvania (2018), we confirmed that the HEG effect was stable under variable environmental conditions. Precipitation for the month of August 2018 in Centre County, Pennsylvania, was 187.2 mm, in contrast to the 65.0 mm rainfall in August of the previous year (National Centers for Environmental Information), producing flooding conditions. Graft progeny again outperformed the control (Fig. 1i and Supplementary Fig. 5). However, all experiments displayed variation in outcomes from graft progeny lines deriving from different parents and graft events (Supplementary Figs. 3–5), implying variation in the molecular events underpinning the HEG process.

### Graft progenies show differential gene expression in enriched pathways

**Fig. 2:**
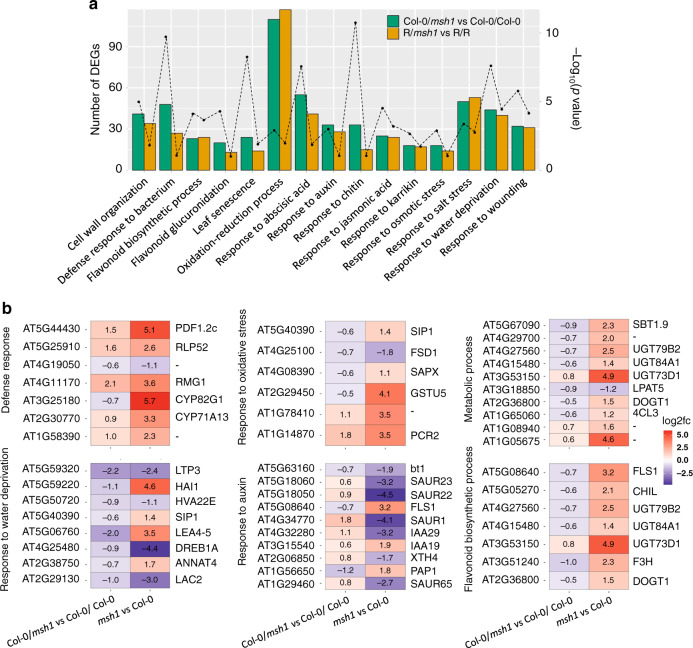
Differentially expressed genes (DEGs) and enriched pathways in graft progenies. **a** Significant enriched GO pathways shared by *Arabidopsis* graft progeny (Col-0/*msh1* vs. Col-0/Col-0) DEGs and tomato graft progeny (R/*msh1* vs. R/R) DEGs. Bar graph represents number of DEGs and dotted line represents −log_10_(*p*-value) for the enrichment test. One-sided Fisher’s exact test was used to compute FDR as implemented in DAVID GO. **b** DEGs from the significant enriched GO pathways of *Arabidopsis* graft progenies (Col-0/*msh1* vs. Col-0/Col-0) and *msh1* mutant (*msh1* vs. Col-0). GO biological process enrichment categories above the cutoff, FDR < 0.01, are shown. DAVID GO was used to conduct the analysis. Source data underlying **a** are provided as a Source Data file.

To discover gene pathways contributing to the enhanced growth phenotype, we first conducted RNA sequencing in graft progenies. Transcriptome analysis of *Arabidopsis* rosette tissues identified 1772 differentially expressed genes (DEGs) in first-progeny generation Col-0/*msh1* (scion/rootstock) compared to Col-0/Col-0 control grafts (Supplementary Data 1). Similarly, R/*msh1* vs. R/R third-generation comparisons in tomato young leaf tissues resulted in 2172 DEGs (1788 ortholog genes in *Arabidopsis*; Supplementary Data 2). Significant enriched Gene Ontology (GO) pathways shared by *Arabidopsis* graft progeny (Col-0/*msh1* vs. Col-0/Col-0) and tomato graft progeny (R/*msh1* vs. R/R) DEG datasets are presented in Fig. 2a, with prolific display of stress and hormone response pathways. We compared DEG datasets from the graft progeny to datasets from rootstock (*msh1* mutant) in *Arabidopsis* to discover significant overlap in shared pathways but with opposite directions of expression changes (Fig. 2b). The gene expression changes observed in *msh1* graft progenies strikingly resembled DEG datasets derived from the Ler × C24 F1 hybrid vigor reported by Wang et al.^19^, revealing a shared emphasis on stress response changes in both heterosis and the HEG effect (Supplementary Fig. 6).

### Graft progeny from *msh1* rootstock experiments undergo methylation repatterning

**Fig. 3:**
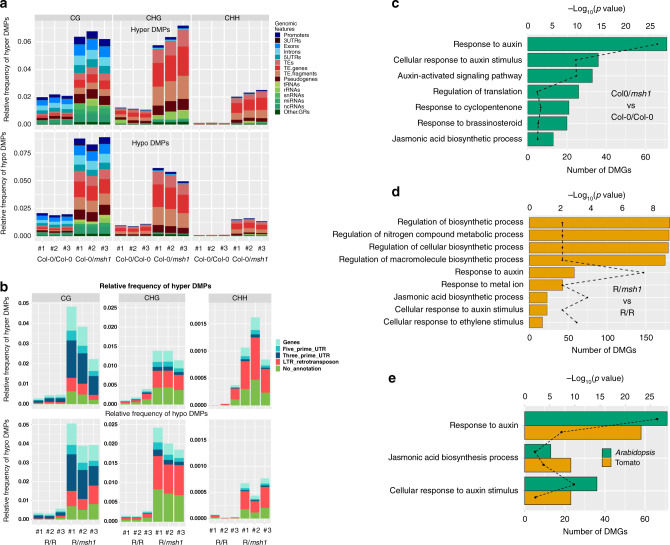
Differentially methylated position genomic distribution and enriched differentially methylated gene networks in graft progenies. **a** The relative frequency of differentially methylated positions (DMPs) in each graft progeny plant over genic regions (blue shades), TE-related regions (red shades), and others (green shades). The average of three control plants (the centroid of Col-0/Col-0 graft progenies) was used as reference. The relative frequency of DMPs in each genomic feature were estimated as the number of DMPs divided by the number of total genomic cytosine positions in each genomic feature. DMP number and frequency of each individual was computed separately and the group means for Col-0/Col-0 graft progenies and Col-0/*msh1* graft progenies are presented. Upper part of the graph shows the distribution of hyper DMPs, the lower part shows the distribution of hypo DMPs. **b** The relative frequency of DMPs in each graft progeny plant over genic regions (blue shades), LTR retrotransposon regions (red shades), and others (green shades). The average of three control plants (the centroid of Rutgers/Rutgers graft progenies) was used as reference. The tomato LTR retrotransposon annotation was acquired by using LTRpred version 1.1.0.^69^ Networks identified by Network Enrichment Analysis Test (NEAT) for the DMGs identified in **c** *Arabidopsis* graft progeny (Col-0/*msh1* vs. Col-0/Col-0); **d** tomato graft progeny (R/*msh1* vs. R/R); **e** three networks shared by both *Arabidopsis* and tomato graft progenies. Bar graph represents number of differentially methylated genes (DMGs) and dotted line represents −log_10_(*p*-value) of the enrichment test. **c**–**e** *p*-values were computed using one-sided hypergeometric distribution test as implemented in NEAT (R package version 1.1.3). Source data are provided as a Source Data file.

To understand the nature of epigenetic changes associated with the HEG phenotype, we conducted whole-genome bisulfite sequencing in the *Arabidopsis* (rosette leaves) and tomato (young leaves) first-generation graft progenies. Of the identified differentially methylated positions (DMPs) in Col-0/*msh1* compared to Col-0/Col-0 control graft, CG context accounted for ∼41%, followed by ∼38% CHH and ∼21% CHG DMPs (Supplementary Fig. 7a). The DMP distribution showed a trend toward hypomethylation for CG and CHG, with the exception of Col-0/*msh1* sample 3 that showed slight CHG hypermethylation, whereas CHH DMPs showed hypermethylation in all cases (Supplementary Fig. 7a). Distribution of DMPs over different genomic features showed the largest portion of DMP variation within TE regions (Fig. 3a).

Trends were similar in tomato, where R/*msh1* DMPs trended toward hypomethylation for CG and CHG and hypermethylation for CHH (Supplementary Fig. 7b). Less robust genome annotation in tomato restricted DMP distribution over different genomic features to genes and untranslated regions (UTRs). However, we used the LTRpred package^20^ to predict linear regression test (LTR) retrotransposons as a component of tomato TE composition. With this annotation, we found that a majority of CHG and CHH DMPs resided within LTR retrotransposon regions, whereas a large portion of CG DMPs overlapped with genes and UTRs (Fig. 3b).

Application of generalized linear regression analysis (GLM) tested significance of the difference between group DMP counts (*msh1* graft vs. control graft) at gene body (transcription start sites (TSS)–transcription end sites (TES)) and promoter regions. For *Arabidopsis*, we used promoter annotation reported by Benhamed et al.^21^ and for tomato, we used the 2 kb upstream region of each gene as promoter in lieu of formal promoter annotation. Genes with a statistically significant difference in DMP counts at gene body and/or promoter were defined as differentially methylated genes (DMGs). With this approach, we identified 3908 DMGs for Col-0/*msh1* vs. Col-0/Col-0 (Supplementary Data 3) and 2681 DMGs for R/*msh1* vs. R/R (Supplementary Data 4). The identified DMGs had substantial numbers of DMPs with a minimum 20% methylation level difference (Supplementary Fig. 8). To screen the DMG datasets for phenotype-related DMGs, network enrichment analysis identified key gene networks present in the dataset. For better comparison between *Arabidopsis* and tomato DMG networks, we used BLAST to identify *Arabidopsis* orthologs of tomato DMGs, resulting in 2215 genes. Network-based enrichment analysis in *Arabidopsis* identified pathways within phytohormone signaling networks, most significant being response to auxin (Fig. 3c). Similarly, five of the nine identified networks were phytohormone-related in tomato, with response to auxin being the most significant. The remaining networks were associated with broader biosynthetic and metabolic processes (Fig. 3d). Cross-species parallel analysis revealed three shared pathways between *Arabidopsis* and tomato, two associated with response to auxin and one with jasmonic acid biosynthesis (Fig. 3e). Consistent with an earlier report of *msh1* epigenetic memory^16^ and other studies^22,23^, these observations infer epigenomic responsiveness of phytohormone networks, particularly as relates directly or indirectly to auxin.

### The graft transmissible *msh1* effect is dependent on RdDM pathway components

Lewsey et al.^10^ demonstrated that sRNAs can transit across graft junctions to affect DNA methylation pattern. We investigated the possible role of mobile sRNA in the HEG effect. However, there were two key distinctions between our study and the Lewsey et al.^10^ report: unlike the earlier study, where hypocotyl grafting (micrografting) was conducted at 7 days after germination, we used the main floral stem for grafting. Second, Lewsey et al.^10^ studied methylation changes in the roots of the grafted plant, whereas we investigated methylation in the seed progenies from the graft plant. These distinctions are important to assessing meiotically transmissible sRNA effects.

**Fig. 4:**
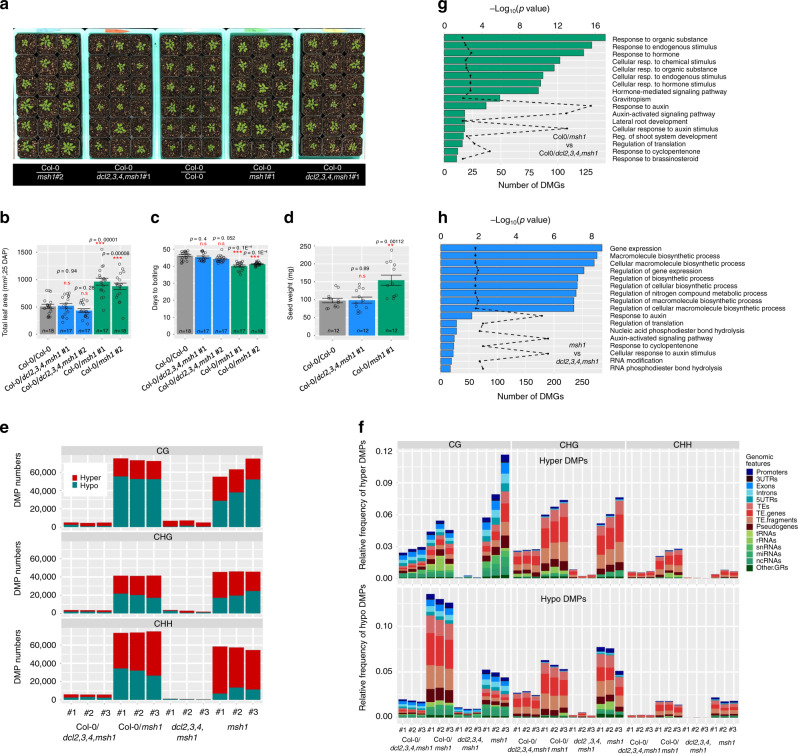
Influence of siRNA on the graft progeny phenotype and methylation repatterning. **a** The phenotype of graft progeny (first generation) from Col-0/Col-0, as graft control, Col-0/*msh1*, with *msh1* rootstock, and Col-0/*dcl2,3,4,msh1*, with a *dcl2,3,4, msh1* quadruple mutant as rootstock. Seedling stage photo was taken at 21 days after planting (DAP). **b** Total leaf area (25 DAP), **c** days to bolting, and **d** seed weight (mg) for the first-generation graft progeny of Col-0/Col-0, Col-0/*msh1* and Col-0/*dcl2,3,4*. **b**–**d** Bars represent means ± SE, *n* represents number of plants in each population. The Mann–Whitney *U*-test with two-sided alternative hypothesis were used to test the significance of the difference of mean in each comparison. **e** Total hyper- and hypomethylation DMP counts of first-generation graft progeny in Col-0/*dcl2,3,4,msh1* vs. Col-0/*msh1* comparison and in rootstocks of *dcl2,3,4,msh1* vs. *msh1* comparison. Each bar graph represents a single plant. CG, CHG, CHH context data are shown separately for each plant. DMPs were defined as hyper if the site methylation difference in comparisons of each individual to the average of reference plants is greater than 0 and defined as hypo if <0. **f** The relative frequency of DMPs over genic regions (blue shades), TE-related regions (red shades), and others (green shades) in first-generation graft progeny in the Col-0/*dcl2,3,4,msh1* vs. Col-0/*msh1* comparison and rootstocks in the *dcl2,3,4,msh1* vs. *msh1* comparison. The centroid of Col-0/*dcl2,3,4,msh1* graft progeny was used as a reference for Col-0/*msh1* analysis and *dcl2,3,4,msh1* was used as reference for *msh1* mutant analysis. Networks identified by Network Enrichment Analysis Test (NEAT) for the DMGs identified are shown for **g** Col-0/*dcl2,3,4,msh1* vs. Col-0/*msh1* and **h** *dcl2,3,4 msh1* vs. *msh1* comparisons. **g**, **h** *p*-values were computed using one-sided hypergeometric distribution test as implemented in NEAT (R package version 1.1.3). Significance codes: NS, no significance, *p < 0.05, **p < 0.01, ***p < 0.001. Source data underlying **b**–**h** are provided as a Source Data file.

To investigate the role of siRNA in the HEG effect, we generated a quadruple mutant by crossing *msh1* and *dcl2,3,4* mutants to prevent 22–24 nt sRNA production^24^. Overall abundance of 23–24 nt siRNA was significantly decreased in *dcl2,3,4,msh1* compared to the *msh1* mutant (Supplementary Fig. 9). We repeated the grafting experiments with Col-0/*dcl2,3,4,msh1*, Col-0/*msh1*, and Col-0/Col-0. Unlike Col-0/*msh1*, the phenotype of graft progeny from Col-0/*dcl2,3,4,msh1* did not display enhanced vigor based on total leaf area, flowering time and total seed weight, resembling Col-0/Col-0 progeny (Fig. 4a–d and Supplementary Fig. 2b). To further assess RdDM dependence of the *msh1* graft effect, we incorporated the methyltranferase mutant *drm2* as scion in grafts to the *msh1* rootstock. In similar effect to the *dcl2,3,4* rootstock results, *drm2* hindered *msh1* graft progeny vigor (Supplementary Fig. 2c) The data appeared consistent with siRNA function in conditioning the graft-derived vigor.

To identify loci contributing to the HEG effect, we compared methylation changes in progeny from Col-0/*msh1* with the Col-0/*dcl2,3,4,msh1* progeny dataset, as well as comparing *msh1* and *dcl2,3,4,msh1* rootstock methylome datasets. These comparisons allowed the identification of RdDM-dependent methylation variation within the *msh1* mutant with the proportion of this variation that is heritable following grafting. Of all identified DMPs in the rootstock comparison, CG accounted for ∼38%, CHG ∼27%, and CHH ∼35%, whereas in the graft progeny comparison, CG and CHH each accounted for ∼39% DMPs and ∼22% CHG (Fig. 4e). Rootstock comparisons also showed a trend in CG DMPs toward hypomethylation, CHG DMPs toward slight hypermethylation (except one sample), and CHH DMPs toward predominant hypermethylation (Fig. 4e). In the graft comparison, CG DMPs were predominantly hypomethylated and CHG DMPs showed a slightly greater trend toward hypomethylation, whereas CHH DMPs showed hypermethylation (Fig. 4e). The largest proportion of DMPs occurred within TE regions in both rootstock and graft comparisons (Fig. 4f).

From these DMP analyses, we identified 3848 DMGs for rootstock (*msh1* vs. *dcl2,3,4,msh1*; Supplementary Data 5) and 2378 DMGs for graft (Col-0/*msh1* vs. Col-0/*dcl2,3,4,msh1*; Supplementary Data 6) datasets. The graft-associated DMG dataset was reduced to 1380 (total minus promoter associated) for more detailed study. In the graft comparison, network-based enrichment analysis identified pathways in phytohormone signaling networks that included response to auxin, response to brassinosteroid, and response to cyclopentanone, as well as lateral root development and gravitropism, likely downstream hormone effects (Fig. 4g). Similar phytohormone pathways, mostly auxin-related, were observed in the rootstock comparison, as well as pathways unique to rootstock that involve gene expression and RNA modification (Fig. 4h). The observed similarities between rootstock and graft progeny methylome behavior are consistent with a model of non-random, targeted changes emerging from rootstock signals to influence graft progeny outcomes.

We investigated the role of sRNA in the methylation repatterning and found 5421 differentially expressed sRNA clusters in rootstock (*msh1* vs. *dcl2,3,4,msh1*; Supplementary Data 7) and 1353 in graft (Col-0/*msh1* vs. Col-0/*dcl2,3,4,msh1*; Supplementary Data 8) progeny analyses, with 501 sRNA clusters shared between them. Analysis of overlap between the total siRNA or *dcl2,3,4*-dependent siRNA loci with DMP datasets indicated that ca. 50% of graft-associated siRNA loci overlap with DMPs, and ~70% of DMPs overlap with siRNA loci, whereas ~15% of DMPs overlap with the *dcl2,3,4*-dependent siRNA loci (Supplementary Fig. 10). These data reflect both direct and indirect influences of siRNA on DMP distribution and appear consistent with previous reports^10^. From the 1380 graft-associated gene-body DMG dataset, we identified 259 DMGs (19%) that overlapped with *dcl2,3,4*-dependent siRNA clusters derived from the rootstock (Supplementary Data 9). As the siRNA and DMP datasets derive from experiments conducted at only one time point in development, and did not include reproductive tissues, it is possible that the remaining indirect associations between *dcl2,3,4*-dependent siRNA clusters and DMGs derive from a different developmental stage in the graft line.

The siRNAs within the RdDM pathway are thought to require high target sequence specificity, so we designed tomato graft experiments that incorporate genetically distinct tomato genotypes as scion and rootstock to possibly obviate *msh1* effect. Under greenhouse conditions, we tested tomato progeny derived from grafting unrelated genotypes to the Rutgers MSH1-RNAi rootstock, with 28 independent grafts involving 8 genetically different cultivars^25,26^, including 3 South American cultivars (LA0134C, LA1162, and LA2285) and 5 Florida elite cultivars (Fla.8872, Fla.8917, Fla.8651, Fla.7804, and Fla.8059) as scion. For each of the grafts, progeny produced no evidence of enhanced growth relative to control grafts (Supplementary Fig. 11). In contrast, 5 independent graft events were carried out with Rutgers as scion on Rutgers MSH1-RNAi as rootstock and all first-generation progeny (26 total) showed growth enhancement. These outcomes support the hypothesis that a level of genetic relatedness is required between rootstock and scion to produce the HEG effect.

### TE methylation repatterning occurs in the rootstock and graft

**Fig. 5:**
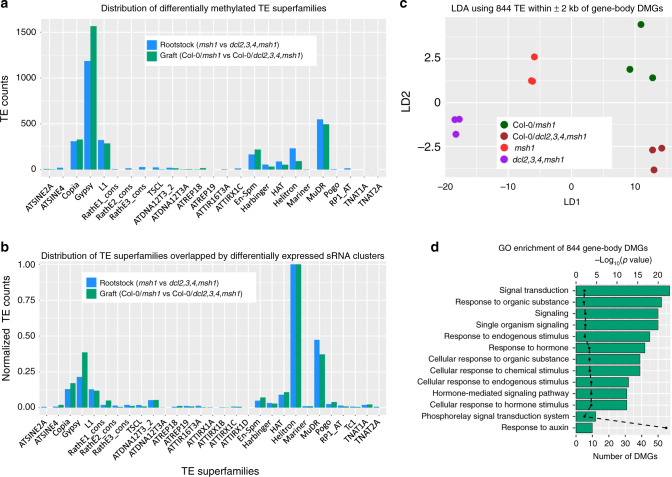
TE methylation repatterning in the *Arabidopsis* graft progenies. **a** Distribution of differentially methylated TE superfamilies in rootstock comparison (*msh1* vs. *dcl2,3,4,msh1*) and graft comparison (Col-0/*msh1* vs. Col-0/*dcl2,3,4,msh1*). **b** Distribution of TE superfamilies overlapping with differentially expressed sRNA clusters in rootstock comparison (*msh1* vs. *dcl2,3,4,msh1*) and graft comparison (Col-0/*msh1* vs. Col-0/*dcl2,3,4,msh1*). **c** Linear discriminant analysis using absolute methylation level of TEs within ±2 kb of gene-body DMGs. **d** Network enrichment analysis (NEAT) of TE-associated (±2 kb) gene-body DMGs. *p*-values were computed using one-sided hypergeometric distribution test as implemented in NEAT (R package version 1.1.3). Source data are provided as a Source Data file.

We examined methylation within TEs, given the large proportion of DMPs located in TE regions, and identified 3112 differentially methylated TEs (DMTEs) in rootstock (Supplementary Data 10) and 3170 DMTEs in graft progeny comparisons (Supplementary Data 11). *Arabidopsis* TE superfamilies such as Gypsy, MuDR, Copia, and L1 were significantly enriched in DMTEs (Fig. 5a). Of these, 2347 (∼74%) DMTEs were shared between rootstock and graft progeny datasets, again confirming that the progeny methylation changes derive from rootstock signals.

Distribution of TE superfamilies that were overlapped by differentially expressed sRNA clusters was similar to the DMTE distribution, with the exception of Helitron elements (Fig. 5b). To examine whether TEs might influence heritable, RdDM-dependent DMGs detected in the graft progeny, we looked for the presence of TEs within (±) 2 kb of identified gene-body DMGs in graft progeny datasets. TE methylation is known to influence the methylation of nearby genes^26–28^. Our analysis showed 844 (∼61%) of the 1380 gene-body DMGs in graft progeny to be associated with TEs. Linear discriminant analysis was used to assess the 844 TEs closest to the gene-body DMGs, indicating that methylation signal at these TEs was sufficient to discriminate the rootstock (*msh1* vs. *dcl2,3,4,msh1*) and graft (Col-0/*msh1* vs. Col-0/*dcl2,3,4,msh1*) datasets (Fig. 5c). This observation again suggests that the gene-proximal TE methylation in graft progenies is RdDM-mediated and impacted by the *dcl2,3,4* mutations.

GO enrichment analysis of the 844 TE-associated DMGs captured 8 of 17 GO pathways found using all graft-associated DMGs (Fig. 5d). A detailed analysis of these DMGs, together with those not directly TE-associated (1380 in total; Supplementary Data 6), revealed RdDM-dependent influences on plant growth. The dataset was populated with significant indicators of signal transduction, auxin transport, and root development regulators, evidence that *msh1* grafting triggers a program that alters root growth. Within the dataset were 86 loci encoding protein kinases and components of protein phosphorylation activity that include *BRL1*, involved in brassinosteroid-mediated regulation of root development^29^, *BAM3*, a receptor-like kinase influencing root meristem growth^30^, and *RPK2*, a modulator of cell proliferation in the root meristem^31^. Another 22 DMGs were involved in the regulation of root development, including *TIR1*, an auxin receptor^32^, *LDL1*, a histone modifier^33^, the auxin-regulated transcription factor *NPH4*^34^, and the receptor-like kinase *CRINKLY4* (*ACR4*)^35^. At least ten identified loci participate in basipetal auxin transport, including *D6 PROTEIN KINASE-LIKE 2* (*D6PKL2*) and *ABCB3*,*6*,*11*,*12*,*14*,*19*, and *20*, a series of ABC B-family transporters of auxin^36^.

Along with these changes, at least 17 DMGs reflected chromatin remodeling that may accompany the graft effects. These genes include DNA methyltransferases *DMT2* (AT4G14140) and *CMT2*, histone demethylase *LDL3*, *CHROMATIN REMODELER* loci *CHR4*, *5*, *11*, and *12*, and the histone regulator *HIRA*. Chromatin remodeler ATPase *BRAHMA* functions in root stem cell maintenance by interacting with PINs that facilitate auxin transport^37^. The cryptochrome *CRY2* participates in primary root elongation^38^, whereas the chromatin remodeler *PICKLE* (*PKL*) is a component of root meristem regulation^39^.

### The *msh1* graft progeny are altered in root phenotype and auxin response

**Fig. 6:**
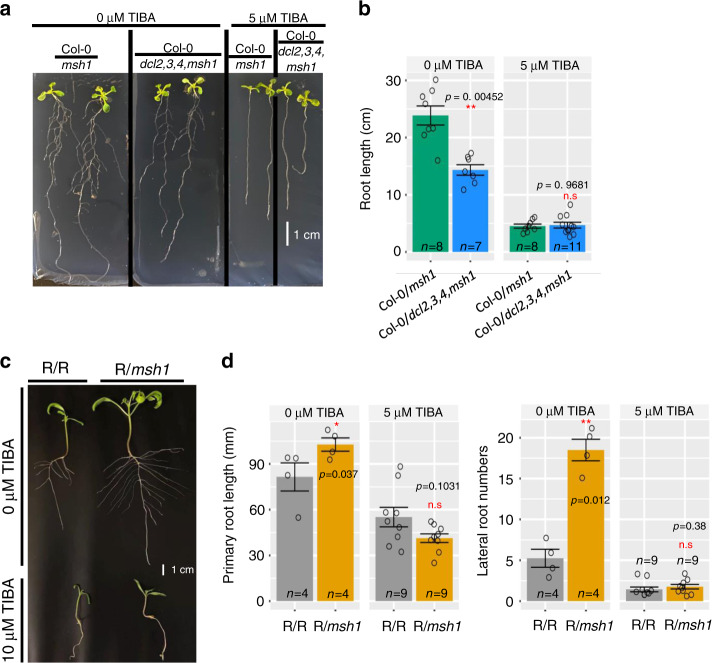
The *msh1* graft progeny root phenotype and TIBA treatment. **a** *Arabidopsis* Col-0/Col-0 and Col-0/*msh1* graft progeny (first generation) growing on 0.5 M MS medium with and without 2,3,5-Triiodobenzoic acid (TIBA, auxin inhibitor) treatment. Photo shows seedlings at 12 days after planting. **b** Total root length (cm), including primary root and all lateral roots of *Arabidopsis* Col-0/Col-0 and Col-0/*msh1* graft progeny with and without 5 μM TIBA treatment. The phenotype for **c** primary root length and **d** lateral root number for tomato Rutgers/Rutgers (R/R) and Rutgers/RNAi*-MSH1*(R/*msh1*) graft progeny (third generation) growing on 0.5 M MS medium with and without TIBA treatment. Photo shows seedling at 19 days after planting. **b**, **d** Bars represent means ± SE, *n* represents number of plants in each population. The Mann–Whitney *U*-test with two-sided alternative hypothesis was used to test the significance of the difference of mean in each comparison. Significance codes: *p < 0.05, **p < 0.01, ***p < 0.001, NS, no significance. Source data underlying **b**, **d** are provided as a Source Data file.

**Fig. 7:**
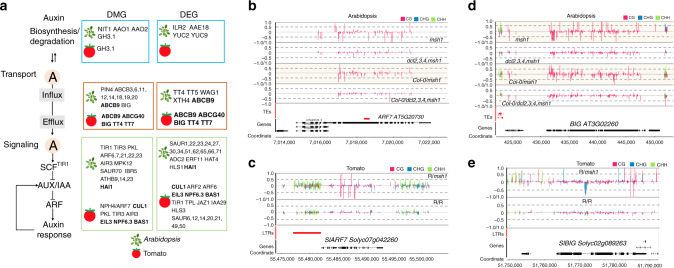
Auxin pathway genes identified in association with the HEG effect. **a** Auxin pathway DMGs and DEGs identified in the *Arabidopsis* Col-0/*msh1* vs. Col-0/*dcl2,3,4,msh1* graft progeny (first generation) comparison and tomato R/R vs. R/*msh1* graft progeny (first generation) comparison. Genes within the blue box are auxin biosynthesis/degradation related, in the red box are transport-related, and in the green box are signaling-related. Genes identified as both DEGs and DMGs are shown in bold. Single cytosine methylation level changes in *Arabidopsis* mutants *msh1* and *dcl2,3,4,msh1*, and graft progenies from Col-0/*msh1* and Col-0/*dcl2,3,4,msh1* (first generation) at genes **b** *ARF7* (AT5G20730) and **d** *BIG* (AT3G02260) loci. Single cytosine methylation level changes in the tomato Rutgers/Rutgers (R/R) and Rutgers/*MSH1*-RNAi (R/*msh1*) graft progeny (first generation) at the **c** *SlARF7*(Solyc07g042260) and **e** *SlBIG* (Solyc02g089263) loci. Methylation level differences at each cytosine were computed by subtracting average methylation level of reference plants from the methylation level of each individual sample. For *msh1* and *dcl2,3,4,msh1* mutants, *dcl2,3,4,msh1* plants were used as reference. For Col-0/*msh1* and Col-0/*dcl2,3,4,msh1* graft progeny, Col-0/*dcl2,3,4,msh1* graft progeny plants were used as reference. Only one plant from each genotype was selected as representative; the pattern differs slightly for different individuals due to fluctuation in methylation. Integrated Genome Browser (version 9.0.2) was used to generate figure. Panel **a** was made by authors based on data from this study and information adapted from Zubo and Schaller^77^.

Auxin is fundamentally important in root growth and development^40,41^, and auxin pathways were consistently prominent in the DMG and DEG dataset analyses in both *Arabidopsis* and tomato. As we also observed root development-related pathways in these datasets, we investigated the root phenotype in *Arabidopsis* and tomato graft progeny. Total root length and lateral root numbers were significantly greater in both *Arabidopsis* and tomato graft progeny seedlings (Fig. 6). Transcriptome analysis of root tissues identified gene pathways with 622 DEGs (Supplementary Data 12), which were similar to those identified in leaf transcriptome assays (Supplementary Fig. 12). Integration of methylome and gene expression datasets revealed a number of loci for auxin transport and signaling that were altered in the HEG effect in both *Arabidopsis* and tomato. However, correspondence of gene expression and methylation changes was more pronounced in tomato than *Arabidopsis* (Fig. 7 and Supplementary Fig. 13). This was also the case in analysis of ABA-related changes in the HEG effect. In the ABA pathway, where the majority of HEG-related gene expression and methylation changes were also associated with signaling, tomato showed much greater correspondence between the methylome and gene expression datasets for these genes (Supplementary Fig. 13). ABA influences lateral root formation in a manner that may be auxin independent^42^.

To assess possible involvement of auxin transport in the altered root phenotype, we included treatment with 2,3,5-triiodobenzoic acid (TIBA), an auxin polar transport inhibitor. Plants grown on MS media containing 5 µM TIBA no longer displayed the enhanced root phenotype when compared to control grafts (Fig. 6), implicating auxin involvement in the altered root phenotype. These observations further the argument for a relationship between methylome effects, where auxin pathway signal was prominent, and the emergent HEG phenotype. The data also imply that altered root growth and architecture contribute to the observed growth vigor.

## Discussion

Grafting is a viable method to improve growth performance of the grafted scion in a broad array of dicot and woody plant species, enhancing fruit quality, production features, and disease resistance^43^. Graft hybridization, for vegetative propagation of the grafted scion, can expand the graft effect to population scale for agricultural gains and possibly influence breeding outcomes^44^. Thus, plant grafting research comprises a robust literature focused predominantly on grafted scion effects but not on heritable (meiotically transmissible) progeny outcomes. To date, investigations of graft effects on seed progeny have been scant. This under-reporting of heritable graft effects may reflect a lack of measurable change in most graft progeny lineages.

Data from this study show a pronounced effect of the *msh1* rootstock in subsequent generations, reflecting sexual transmission of the derived changes. *Arabidopsis* graft studies included second-generation growth and first-generation methylation changes, but the tomato studies include up to the fifth generation lines following grafting (third generation for methylome studies). The similarity in *Arabidopsis* and tomato molecular data, together with observed heritability of tomato field effects, demonstrate that graft methylation repatterning effects can be heritable over more than a single generation.

*Arabidopsis* and tomato species represent plant systems with annual life cycles and transmission through gametogenesis of epigenetic effects may be necessary to condition the plant vigor outcomes that we observed. RdDM is known to participate in significant epigenetic reprogramming during reproductive development^45^. The process is essential to establishing distinct methylation profiles in egg and central cells of the female gametophyte^46^, as well as in vegetative and sperm cells of the male gametophyte^47,48^. However, the *msh1* effect could differ in perennials. Perennial or woody systems rely on annually recurrent activity of the vascular cambium, a complex meristem tissue for secondary growth^49^, which also likely implements RdDM-mediated changes^50,51^. Therefore, it is possible that *msh1* graft effects could manifest in scion secondary growth of the graft individual.

Mutation of *MSH1* triggers significant changes in DNA methylation and expression of stress-related pathways^17,52^. In *msh1* self-crossed progeny, RdDM-mediated changes trigger transgenerational, nongenetic stress memory^16,17^, yet produce heritable growth vigor in graft progeny. Both *msh1* memory and graft progeny methylation patterns display similarities with the parental *msh1* reprogrammed methylome within targeted genomic regions, including auxin-related pathways (Fig. 7). Intersection of common pathways between memory and grafting outcomes include phytohormone signal transduction, RNA processing, and circadian clock effects^16^, consistent with sharing an *msh1* lineage. Assessment of shared candidate loci targeted for methylation repatterning in the *msh1* mutant, memory, and graft progeny datasets included, e.g., *ARF7*, involved in lateral root formation^53^, *BIG*, involved in polar auxin transport^54^, and *GIGANTEA*, a central regulator of the circadian clock network^55^ (Fig. 7 and Supplementary Fig. 12). The substantial number of altered pathways shared in *msh1* rootstock and graft progeny suggests that scion behavior is less important than *msh1* rootstock effects in directing graft progeny methylome outcomes and assaying the *msh1* rootstock could prove predictive of graft outcomes.

Of the 1380 *dcl2,3,4*-dependent DMGs identified in graft progeny, ~19% (259) overlapped with *dcl2,3,4*-dependent siRNA clusters identified in the *msh1* rootstock. Similar to the DMG dataset, these putative siRNA targets consisted of several loci involved in phytohormone response, including the ethylene signaling component *EIN2* and auxin response and transport loci *ARF21*, *ALA11*, *BIG*, and *ABCB6*. Other putative direct targets included chromatin remodeling and light response components *CHR5*, *PKL*, and circadian clock regulator *GI*. The remaining 81% “indirect” siRNA-targeted (*dcl2,3,4*-dependent) DMGs may represent RdDM effects within grafted tissues that take place during reproduction in the graft^45^, a stage that was not included in our assay. The compelling association between rootstock and derived graft progeny methylome behavior was similarly evident in 74% of DMTEs shared between these two datasets.

Graft progeny outcomes in both *Arabidopsis* and tomato showed evidence of altered ABA response in both gene expression and methylation repatterning datasets (Supplementary Fig. 14). Numerous shared genes involved in ABA signaling were altered in both *Arabidopsis* and tomato, but the correspondence between methylome and transcriptome datasets was more pronounced in tomato. This observation may be the consequence of more robust DEG signal in young tomato leaves than in the more heterogeneous *Arabidopsis* rosette tissue sampled for these experiments and suggests that tomato may offer a more informative model than *Arabidopsis* for studies of DEG–DMG intersection.

Experiments in tomato showed that a degree of genetic relatedness between scion and rootstock may be necessary for enhanced vigor in the graft progeny. In contrast to numerous reports of phenotypic variation arising in scions after grafting to inter-specific and intergeneric rootstocks^11,12,43,56^, we observed no enhanced vigor effect in progeny of tomato grafts when we included distantly^25^ related cultivated tomato genotypes as scion. A likely explanation for this outcome is that constraints exist for siRNA-target gene sequence homology^57^. Tomato has been shown to incorporate significant TE-related variation within their diverse genotypes^26^. However, our study focused only on the Rutgers rootstock and will need to expand to a broader rootstock collection for fuller elaboration of graft genetic distance effects. Our observations to date, including heritability of *msh1* graft effects, lead us to conclude that the *msh1* graft phenomenon is distinct from previously described agricultural graft systems.

The increased progeny vigor that derives from *msh1* grafting may reflect an epigenetic influence on plant fitness. Previous reports have implicated epigenetic effects in the expression of heterosis or F1 hybrid vigor^19,58^. Heterosis studies in *Arabidopsis* reveal altered expression of stress^19^ and auxin response^59^ pathways that are similar to the *msh1* HEG effect. Circadian clock components, regulated epigenetically, also contribute to heterosis^60^. Hybrid vigor is speculated to be a function of energy allocation through a balance of metabolic and stress response processes^59,61,62^. Methylome analysis of *msh1* graft progeny identified numerous metabolic (sugar transport and growth), RNA processing and stress response components showing siRNA-dependent differential methylation. The *msh1* mutant, memory, and graft progeny display changes in circadian clock and light response factors and auxin-related pathways, together with other phytohormone effects, as primary targets^16,17,39^. Consistent with this model of a metabolic and stress poise to condition the enhanced vigor phenotype, a particularly interesting RdDM-dependent DMG in graft progeny is *TOR*, a central growth regulator that mediates this balance^63^ and interacts with auxin to influence growth^64^.

Resolution of gene pathways through methylome analysis was accomplished by incorporating *dcl2*, *dcl3*, and *dcl4* mutants to identify siRNA-dependent methylation variation. The approach was coupled with high resolution methylation analysis by the Methyl-IT platform^65^. As in earlier studies of *msh1* mutant and memory effects^16^, and in other biological systems^66^, integration of gene expression and methylome data provided greater resolution than either dataset alone. We assume that this enhanced resolution of potentially central gene pathways through detailed methylome analysis is a consequence of greater spatio-temporal stability of methylation signal than gene expression signal in plants when multiple cell types are pooled for analysis^67^. Still, it was feasible to detect a relationship between gene expression and methylation variation by implementing a Linear-by-Linear Association and Mann–Whitney test. It is reasonable to assume that transcriptome changes within gene pathways that display RdDM-dependent differential methylation likely involve low magnitude or spatially regulated changes in expression to effect quantitative variation in phenotype.

Auxin response pathways and visibly enhanced root growth in the *msh1* graft progeny appear to contribute directly to the HEG effect. These outcomes were seen in both *Arabidopsis* and tomato, and similarly enhanced growth in *msh1*-grafted soybean progeny was previously shown to be associated with altered auxin pathway expression^68^, reinforcing the argument that *msh1* effects are programmed, and the epigenetic influence on phytohormone pathways is conserved.

It was not feasible in this study to discriminate site-directed gene-body methylation changes from those deriving from neighboring TE sequences. Previous studies by others have shown that TE-associated methylation changes can impact nearby gene expression^27,28^, perhaps sustaining local methylation transgenerationally. In tomato, a class of TEs displays stress responsive behavior and influences plant phenotype^69^. Much of the relatively subtle, gene-associated methylation repatterning reported here could arguably go undetected by most methylation analysis programs or be attributed to methylome drift. However, incorporation of the siRNA-null mutants in the analysis of graft progeny provided resolution of the graft-directed methylation changes to confirm RdDM influence.

Although we employed methylome analysis to document heritable epigenomic changes following grafting, cytosine methylation comprises only one chromatin feature altered coordinately in response to stress. Activity of FORGETTER1, following heat stress, directs changes in stress-associated reorganized chromatin states that are sustained and heritable^70^. In the model *Caenorhabditis elegans*, it is possible to demonstrate transgenerational stability of histone modifications as repressive marks^71^, as well as germline-to-soma communication of stress memory^72^. In plants, this type of heritable epigenetic influence on plant environmental responsiveness could offer potential for future agricultural improvement strategies^73–75^. In the case of the *MSH1* effect, field demonstration of heritable growth vigor and yield gains in tomato are an important confirmation of how close these applications might be to feasible implementation.

## Methods

### Plant materials and growth conditions

For *Arabidopsis* (*Arabidopsis thaliana*) used in the study, clean seeds were sown on peat mix in square pots, with stratification at 4 °C for 2 days before transport to growth chambers (22 °C, 12 h DL, 120 − 150 μmol m^−2^ s^−1^ light). *Arabidopsis* *dcl2-1/3-1/4-2* (CS16391) triple mutant seeds were obtained from ABRC Stock Center and were crossed to *msh1*, to obtain *dcl2,3,4,msh1* quadruple mutant. Primers used for screening mutant plants are listed in Supplementary Table 1.

For tomato (*Solanum lycopersicum* L.), MSH1-RNAi suppression lines in the cv. Rutgers background were developed previously^18^ and progeny from two independent transformation events (T17 and T20) were used in grafting experiments. Plants in the greenhouse were germinated on MetroMix 200 medium (SunGro) and maintained at 26–28 °C with a 15 h day length and at 20–22.8 °C with a 9 h dark period. For the inter-cultivar grafting experiment, seed of elite tomato cultivars Fla.8872, Fla.8917, Fla.8651, Fla.7804, and Fla.8059 are from the University of Florida breeding program and seeds of South American cultivars LA0134C, LA1162, LA2285 were obtained from the Tomato Genetics Resource Center at U.C. Davis.

### Graft experiments

For *Arabidopsis*, grafts were generated as described previously^17^. Briefly, wedge-cleft grafting was performed with primary inflorescence stems 5–10 cm above rosettes. Parafilm was used to secure the wedge grafts, maintain scion-rootstock contact, and prevent desiccation. Grafted plants were kept in a mist chamber for 1–2 weeks until scions showed growth, then grafted plants were acclimatized to normal growth conditions. Non-grafted floral shoots were removed to promote growth of the primary grafted floral stem. Each grafted scion was harvested separately, giving rise to generation-one progeny. This process generated Col-0/Col-0, Col-0/*msh1*, and Col-0/*dcl2,3,4,msh1* grafts (scion/rootstock). For tomato, wedge grafting was carried out with seedlings at the two- to four-leaf stage following the procedure described in Yang et al.^18^.

### Tomato yield trials

In 2017, a single-location trial was conducted by a third-party company in Thonotosassa, FL. Graft progeny generations 1, 2, and 3 were transplanted on 27 February 2017 and the final plant evaluation was taken on 15 June 2017. Plants were spaced 19 inches apart in raised, plastic-covered beds with 6 feet between each bed for a final planting density of 4585 plants per acre. The soil had a pH of 6.4, a cation exchange capacity of 4.0 meq per 100 g soil, and 2.1% organic matter. The soil texture consisted of 99.5% sand, 0% silt, and 0.5% clay particles. Average plot size was between 21 and 27 plants, and yield evaluation was taken from the inner 12 plants. Total of 13 entries spanning generations 1, 2, and 3 after the graft were replicated three times in a randomized complete block design (RCBD). A standard fertility program was used for the area and soil type for optimum yields. Yield harvest data were reported on a total fruit count and weight per plot basis. Tomato yellow leaf curl virus infection was evaluated on a scale of 0 to 10, where 0 indicated that there was no sign of infection and 10 indicated that the infection was severe on 15 June 2017.

In 2018, a single-location trial was conducted by a third-party company in Huasna, CA. Graft progeny generation-4 seedlings were transplanted on 19 March 2018 and the final plant evaluation was taken on 7 September 2018. Plants were spaced 18 inches apart in raised beds with 6.66 feet between each bed for a final planting density of 4360 plants per acre. The soil had a pH of 7.4, a cation exchange capacity of 23 meq per 100 g soil, and 2.5% organic matter. The soil texture consisted of 37% sand, 29% silt, and 34% clay particles. Plot size was defined as 15 plants and yield evaluation was taken from the inner 11 plants. Total of three generation-4 entries were replicated six times in a RCBD. A standard fertility program was used for the area and soil type for optimum yields. Yield harvest data were reported on a total fruit count and weight per plot basis. The Rutgers/Rutgers control packet had contaminating Money Maker wild type (WT) seeds present. Each plot was scored for the presence of Money Maker plants and yield was adjusted to correct for excess yield of Money Maker over Rutgers. Money Maker was present in the same trial for a separate study, so a correction calculation could be properly made.

In 2018, a single-location trial was conducted at the Russell Larson Research and Education Center in Pine Grove Mills, PA. Seeds were sown under greenhouse conditions on 2 April 2018 and transplanted on 31 May 2018. Plants were spaced 18 inches apart in raised, plastic-covered beds with 6 feet between each bed. Plot size was defined as 20 plants and yield evaluation was taken from the inner 14 plants. Total of 31 entries, including graft progeny generations 2, 3, and 5 were replicated six times in a RCBD. A standard fertility program was used for the area and soil type for optimum yields. Three harvests were performed in the weeks of 27 August, 3 September, and 10 September 2018. Yield harvest data were reported on a total fruit count per plot basis. Development of WT/MSH1 and WT/WT graft progenies did not involve a phenotypic selection process. For each generation, the seed from each genotype and plot were collected, pooled, and used for the subsequent field trial without considering previous trial performance.

Statistical analysis for the tomato field data involved linear mixed model *y*_*ij*_ ∼ genotype_*i*_ + block_*j*_ + *e*_*ij*_, where genotype_*i*_ is treated as a fixed effect and block_*j*_ is treated as a random effect, and *e*_*ij*_ is the residual error, implemented by lmerTest R package (version 3.1-2). Tests for significant differences in line means were performed using linear mixed hypothesis tests with the lmer function from the same package.

### RNA sequencing and analysis

For *Arabidopsis*, *msh1* and *dcl2,3,4,msh1* quadruple mutant and graft progenies were grown in parallel, and three plants from each line were selected for sequencing. Whole rosettes at bolting stage were flash frozen and ground in liquid nitrogen. For tomato, young leaves of 6-week-old R/*msh1* and R/R control graft progenies were flash frozen and ground in liquid nitrogen.

For all samples, a portion of ground tissue was processed with the DNeasy Plant Kit (Qiagen, Germany) for genomic DNA with RNA removal according to the manufacturer’s protocol for subsequent bisulfite sequencing. The remaining portion of ground tissue was used for RNA extraction, including DNA removal, using NucleoSpin RNA Plant Kit (Macherey-Nagel, Germany). sRNA extraction used the NucleoSpin miRNA Plant Kit (Macherey-Nagel, Germany) following the manufacturer’s protocol.

Whole-genome bisulfite sequencing was conducted on the Hiseq 4000 or HiSeq X-ten analyzer (Illumina, USA) at BGI-Tech (Shenzhen, China), according to manufacturer’s instructions. Genomic DNA was sonicated to 200–300 bp by Covaris and the fragmented DNAs were tested by Gel-Electrophotometrics and purified with the MiniElute PCR Purification Kit (Qiagen, Germany), and incubated at 20 °C after adding End Repair Mix. DNA was purified, a single “A” nucleotide was added to the 3′-ends of blunt fragments, sample was re-purified, and methylated adapter was added to 5′- and 3′-ends of each fragment. Fragments of 300–400 bp were purified with the QIAquick Gel Extraction Kit (Qiagen, Germany) and subjected to bisulfite treatment with the Methylation-Gold Kit (ZYMO), followed by PCR and gel purification (320–420 bp fragments selected). Qualified libraries were paired-end sequenced on the Xten analyzer for *Arabidopsis* and on the Hiseq 4000 for tomato (150 bp read length and at least 4 Gb data per sample for *Arabidopsis* samples and at least 20 Gb per sample for tomato samples).

### Methylation analysis

Raw sequencing reads were quality-controlled with FastQC (version 0.11.5), trimmed with TrimGalore! (version 0.4.1) and Cutadapt (version 1.15), then aligned to the TAIR10 reference genome using Bismark (version 0.19.0) with bowtie2 (version 2.3.3.1). The deduplicate_bismark function in Bismark with default parameters was used to remove duplicated reads and reads with coverage >500 were also removed to control PCR bias. Whole-genome bisulfite conversion rate was computed based on chloroplast genome read counts for every sample, with conversion rate >99% for all samples. DMPs were identified using Methyl-IT (version 0.3.2; https://github.com/genomaths/MethylIT) R package as described previously^16^ with some parameters modified for this study. Briefly, cytosine with minimum read coverage of 4 and minimum methylated reads of 3 were used. Hellinger Divergence (HD) was calculated with a pool of control (wild type) samples as reference. Cytosines with methylation level difference >20% in the treatment vs. reference comparison were selected and further filtered by estimating the optimal cutoff for HD based on Youden index to obtain DMPs.

To identify DMGs in *Arabidopsis*, we selected loci with at least seven DMPs and DMP density of 0.0003, then carried out group comparison using LTR to select loci with log_2_fold change >1 and adjusted *p*-value < 0.05 (Benjamini and Hochberg method). For gene body (TSS–TES) DMGs, we used TAIR10 version 38 GTF annotation. For promoter DMGs, we used annotation from Benhamed et al.^21^. For TEs, we used annotation from Jin and Hammell^76^. A similar approach was used in tomato, where the Tomato ITAG3.0 genome was used for annotation. NCBI BLASTP was used to identify the homolog of tomato genes in *Arabidopsis*, the detailed code is available at https://github.com/genomaths/genomaths.github.io/tree/master/blastp.

For principal component plus linear discriminant analysis of 844 TE-associated gene-body DMGs, we applied prcomp (implemented in Methy-IT function pcaLDA) from the R package stats (version 3.6.0). The sum of absolute methylation level over each TE was used for the PCA-LDA. A complete documentation of the analysis (with Methyl-IT) is available at https://genomaths.github.io/.

We conducted analysis of gene expression related to methylation data. For each DEG, the group average of the density of methylation levels on gene body (plus 1 kb up and downstream) was computed as described in Yang et al.^16^ and shown in Methyl-IT documentation at https://genomaths.github.io/methylit/index.html.

### RNA sequencing and analysis

For both *Arabidopsis* and tomato, RNA libraries were constructed as described in the TruSeq RNA Sample Preparation v2 Guide. For *Arabidopsis*, libraries were sequenced with the 150 bp read, paired-end option, in the Novaseq analyzer (Illumina, USA) at BGI-Tech (Shenzhen, China) and for tomato, with the High Output, 75 bp read, paired-end option (at least 60 million reads per sample) in a NextSeq analyzer (Illumina, USA) at the Penn State University Huck Institute genomics core facility. Raw sequencing reads were quality-controlled, trimmed with TrimGalore! (version 0.4.1). Trimmed reads were then aligned to the TAIR10 reference genome using using STAR (version 2.7.3a) with –twopassMode = Basic and –outFilterMultimapNmax = 1 parameters, retaining only uniquely mapped reads. The read count data were generated from the BAM files by using QoRTs software package (version v1.3.0) with –minMAPQ = 25 option. edgeR package (version 3.26.8) was used for gene count normalization and to identify DEGs (adj.*p*Val ≤ 0.1, |log_2_FC| ≥ 0.5). DAVID Bioinformatics Resources 6.8 was used for GO function enrichment analysis. GO terms with Fisher’s exact *P*-value ≤ 0.1 and gene counts ≥ 10 in each GO were selected.

### sRNA-seq and analysis

sRNA-seq libraries were constructed using BGI’s BGISEQ-500 Small RNA Library protocol (document number SOP-SS-045) and sequenced on a BGISEQ-500 with a single-end 50 bp run length. Small RNA-seq data were aligned to the *A. thaliana* genome assembly (version TAIR10) using ShortStack (version 3.8.3) with default parameters, except that the “alignonly” switch was activated. Each library was individually aligned, followed by merging the resultant bam files using SAMtools merge. edgeR package (version 3.26.8) was used to identify differentially expressed sRNA clusters (adj.*p*Val ≤ 0.1, |log_2_FC| ≥ 0.5).

### TIBA treatment

Graft seeds were surface sterilized in 20% (v/v) sodium hypochlorite and rinsed thoroughly with sterile water four times. Seeds were sown on 0.5 M Murashige and Skoog medium (Sigma, USA) supplemented with 1% (w/v) agar 0 (control, dimethyl sulfoxide (DMSO)) or 5 µM 2,3,5-triiodobenzoic acid (Sigma, USA; catalog number T5910) dissolved in DMSO. Square (10 × 10 cm) petri dishes (VWR, USA) were used for *Arabidopsis*. Following plating, seeds were stratified at 4 °C for 2 days, then moved to a growth chamber under 12 h daylight and 120–150 μmol m^−2^ s^−1^ light intensity. *Arabidopsis* roots were imaged after 12 days of plating on the growth medium and total root length was measured using ImageJ (https://imagej.nih.gov/ij/index.html). For tomato, a similar procedure was used, except that 2,3,5-triiodobenzoic acid treatment concentration was 10 µM and the growth containers were 8 oz clear cups (Fabri-Kal, USA).

### Reporting summary

Further information on research design is available in the Nature Research Reporting Summary linked to this article.

## Supplementary information

Supplementary Information

Peer Review File

Reporting Summary

Description of Additional Supplementary Files

Supplementary Data 1-12

## Data Availability

Data supporting the findings of this work are available within the paper and its Supplementary Information files. The datasets generated and analyzed during the current study are available from the corresponding author upon request. All next-generation sequencing data generated by this study were deposited to Gene Expression Omnibus database with the primary accession code GSE152570. *Arabidopsis* and tomato genome used as reference are available at http://ensemblgenomes.org/.  Source data are provided with this paper.

## Source data

Source Data

## Code availability

All of the customized code used in this study, including methylation analysis with Methyl-IT, DMP classification based on signal detection, principal component and linear discriminant analyses, and BLAST homolog gene analysis are available at GitHub^78^.
