# Supplementary material for: MSH1-induced heritable enhanced growth vigor through grafting is associated with the RdDM pathway in plants
Source: Nat Commun. 2020 Oct 22;11:5343. doi: 10.1038/s41467-020-19140-x (PMC7582163; doi:10.1038/s41467-020-19140-x)
Supplement: Supplementary file 1 — Supplementary Information [file 41467_2020_19140_MOESM1_ESM.pdf]

MSH1-induced heritable enhanced growth vigor through grafting is associated with the RdDM pathway in plants

Kundariya *et al.*

Supplementary Table 1. Primers used in the study for mutant identification.

Primer name	Sequence (5'-3')
MSH1-F	ACGGAAAAAGTTCTTTCCAGG
MSH1-R	GCTTTCCATCGGCTAGGTTAG
Sail LB3	TAGCATCTGAATTTCATAACCAATCTCGATACAC
DCL2-1-RP	CTTCACAGGAGTTTTTGGCTG
DCL2-1-LP	TGAATCATCTGGAAGAGGTGG
DCL3-p1	CTGAATATGGATAATAAGTTTGAGACATATC
DCL3-p2	GGACTCAATGCAATATAGAGCTTT
Salk_LBb1.3	ATTTTGCCGATTTCGGAAC
DCL4-2-RP	TTTGCCAGTCTTACAAGTGGG
DCL4-2-LP	CAGAAGAGCAATCGAAGAGAACTT
Gabi-8474	ATAATAACGCTGCGGACATCTACATTTT

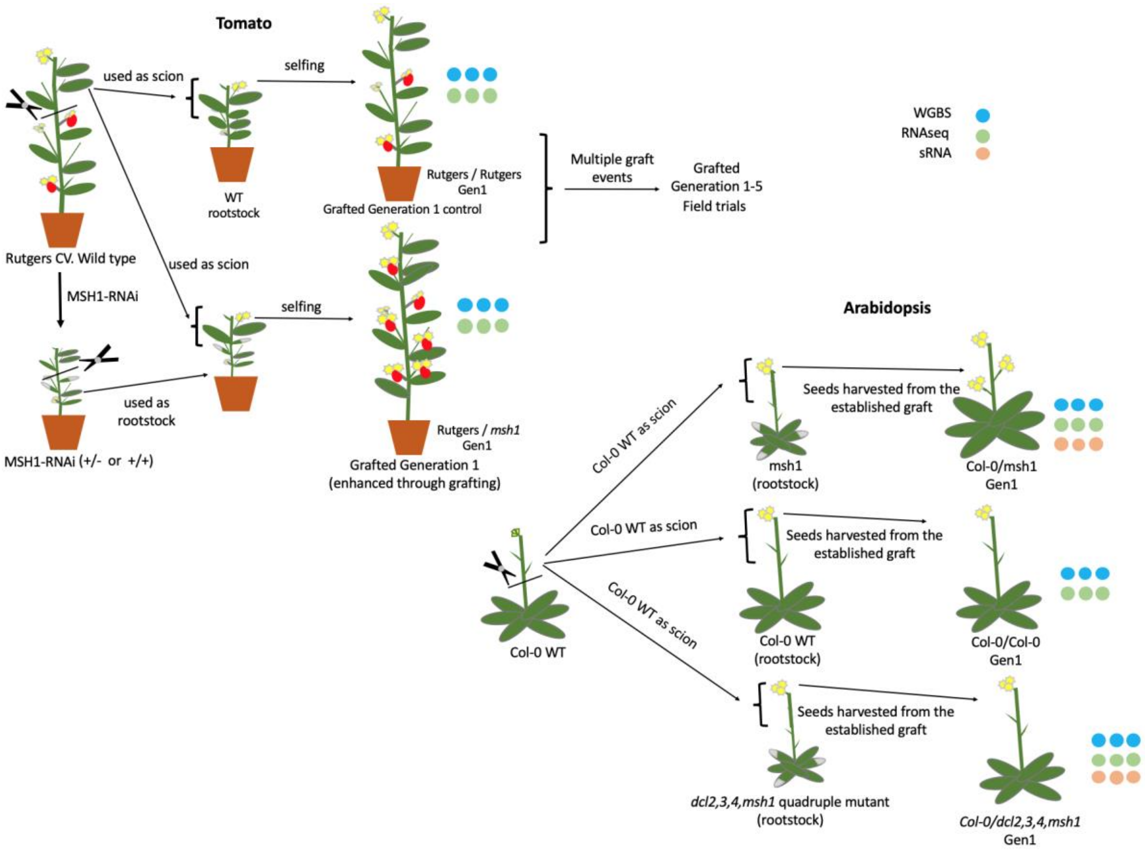

Supplementary Fig. 1. Graft experimental design and sample collection strategy. The graft progenies were created by combining wild type (Arabidopsis Col-0; tomato cv. Rutgers) scions to *msh1* mutant (Arabidopsis) or RNAi knockdown (tomato) rootstocks. Grafts were conducted at flowering stage. In Arabidopsis, the *dcl2,3,4,msh1* quadruple mutant was also included as rootstock. Control grafts included wildtype rootstocks. Three plants from each graft progeny lineage were processed for bisulfite sequencing (blue dots), RNAseq (green dots) and sRNA (orange dots in Arabidopsis).

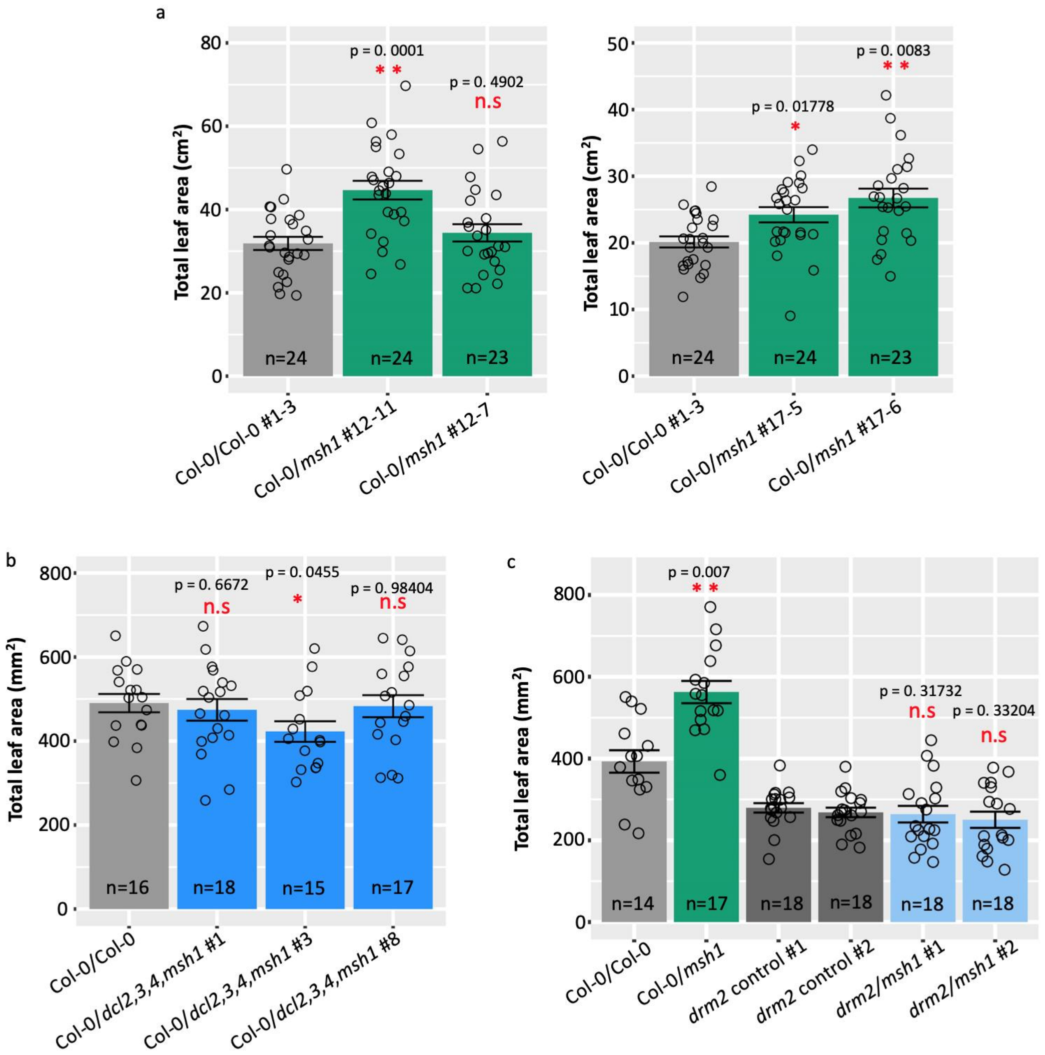

Supplementary Fig. 2. Additional Arabidopsis grafting experiments. a, Total leaf area (35 DAP) of generation 2 graft progenies coming from two independent Col-0/*msh1* grafts. Mean of Col-0/*msh1* was compared to Col-0/Col-0 to obtain p-value. **b**, Total leaf area (20 DAP) of addition Col-0/ *dcl2,3,4,msh1* grafts. Mean of Col-0/*dcl2,3,4,msh1* was compared to Col-0/Col-0 to obtain p-value. **c**, Total leaf area (19 DAP) of Col-0/Col-0, Col-0/*msh1*, *drm2* (controls), and *drm2/msh1*. Col-0/Col-0 and Col-0/*msh1* grafts in this panel are the same as shown in Fig. 1c. Mean of Col-0/*msh1* was compared to Col-0/Col-0 to obtain p-value, while mean of *drm2/msh1* was compared to the *drm2* control to obtain p-value. For panels **a-c**, Bars represent means ± SE, n represents number of plants in each population. The Mann-Whitney U test with two-sided alternative hypothesis was used to test the significance of difference of mean in each comparison. Significance codes: n.s, no significance, * (p < 0.05), ** (p < 0.01), *** (p < 0.001). Source data are provided as a Source Data file.

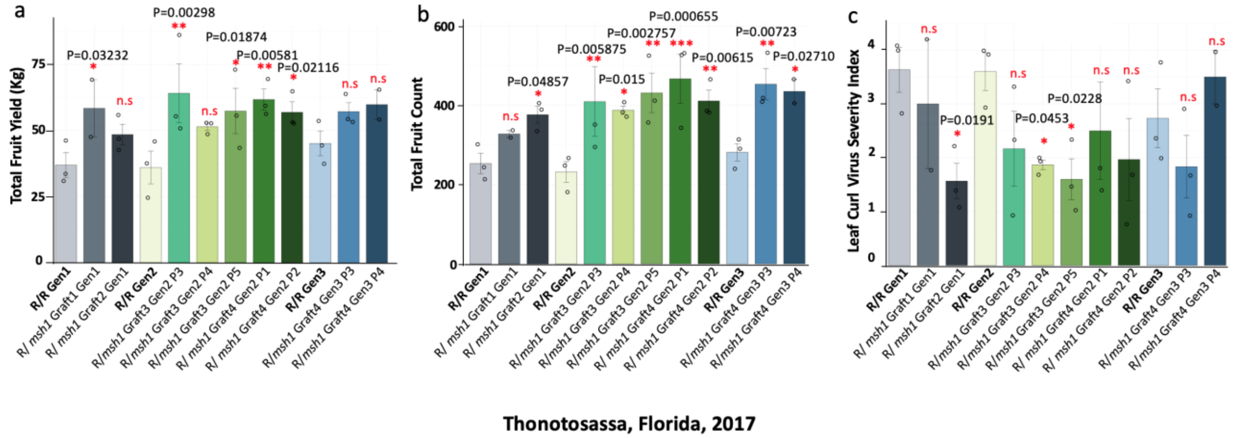

Supplementary Fig. 3. Tomato Florida 2017 field trial data. **a**, Total fruit yield (kg), **b**, total fruit number and **c**, leaf curl virus severity index of tomato graft progeny (the first, second and third generation) for Rutgers/Rutgers and Rutgers/*msh1* grafting events in the 2017 Florida field experiment. Graphs show the average per plot, with each plot represented by a single dot and each bar graph representing the mean of plants from the same parental plant. Error bars represent means ± se (n=3). Significance codes: * (p < 0.05), ** (p < 0.01), *** (p < 0.001) for significance level of Wald tests (each Rutgers/*msh1*group compared against its corresponding Rutgers/Rutgers control in the same generation). Tests for significant differences in line means were performed using linear mixed hypothesis as described in the Method section, implemented by the *lmer* function from R package “lmerTest”. Source data are provided as a Source Data file.

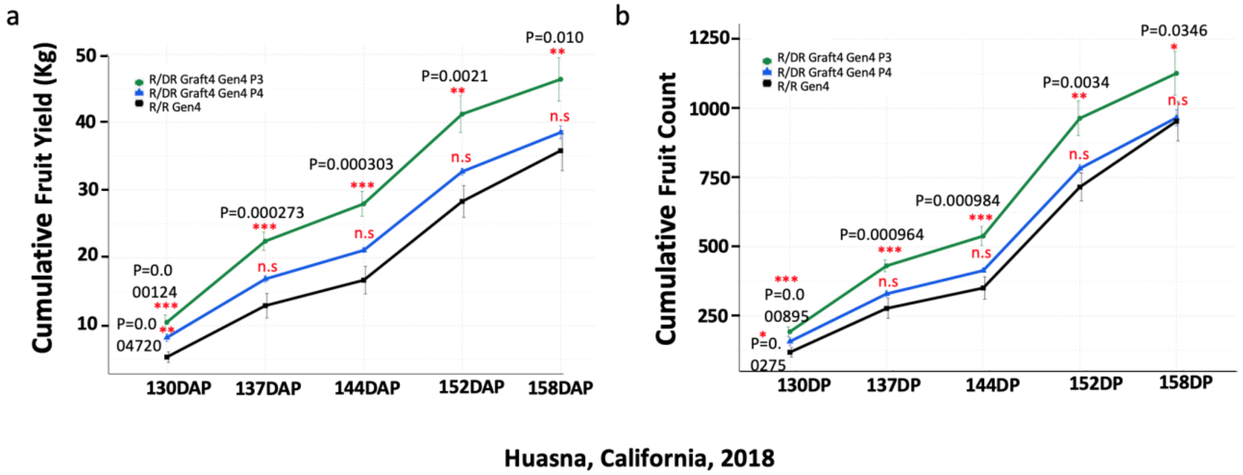

Supplementary Fig. 4. Tomato 2018 California field trial data. **a**, Cumulative total fruit yield (kg) and **b,** fruit counts for tomato fourth-generation graft progeny for Rutgers/Rutgers and Rutgers/*MSH1-RNAi* grafts. Graphs show the average total fruit number per plot at 5 time points (130-158DAP). Error bars represent means ± se (n=6). Each line represents progeny data from different parental lines. Significance codes: * (p < 0.05), ** (p < 0.01), *** (p < 0.001) for significance levels of the Wald test (each Rutgers/*msh1*group compared against its corresponding Rutgers/Rutgers control). Tests for significant differences in line means were performed using linear mixed hypothesis as described in the Method section, implemented by the *lmer* function from R package “lmerTest”. No significant difference was observed among the average 100 seed count weights of R/R (0.16 gm) and R/DR (graft 4 P3 at 0.13 gm and graft 4 P4 at 0.14 gm) indicating that the increased seedling vigor does not derive from seed quality. Source data are provided as a Source Data file.

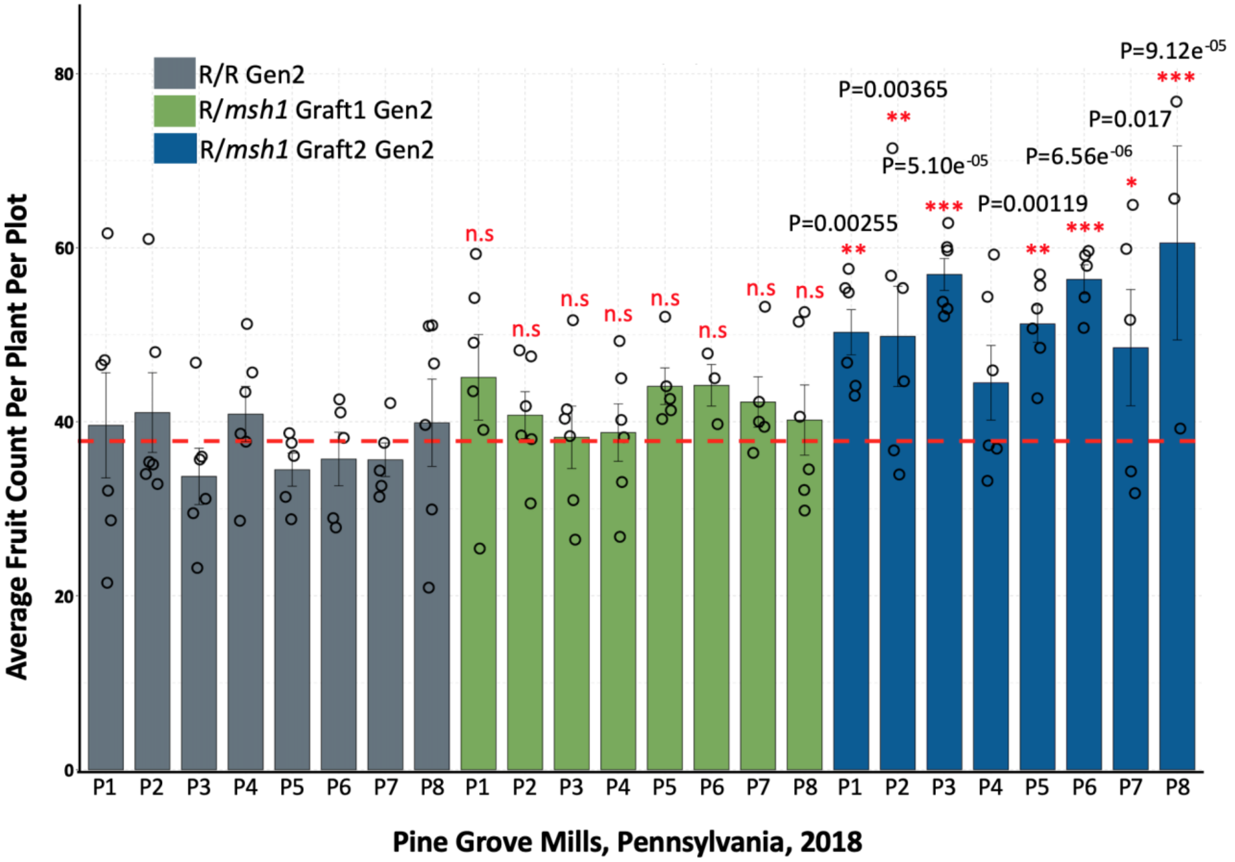

Supplementary Fig. 5. Tomato 2018 Pennsylvania field trial data. Total fruit number per plant for tomato graft progeny (second-generation seed was collected from the 2017 Florida trial from individual gen1 plants). Graph shows the average total fruit number per plant per plot, with each plot represented by one dot. Error bars represent means ± se (n=6) of plants from the same parental plant. Significance codes: * ($p < 0.05$), ** ($p < 0.01$), *** ($p < 0.001$) for significance level of the Wald test (each Rutgers/*msh1* group compared against the mean of all Rutgers/Rutgers control plants, indicated by the red dash line). Tests for significant differences in line means were performed using linear mixed hypothesis as described in the Method section, implemented by the *lmer* function from R package “lmerTest”. Source data are provided as a Source Data file.

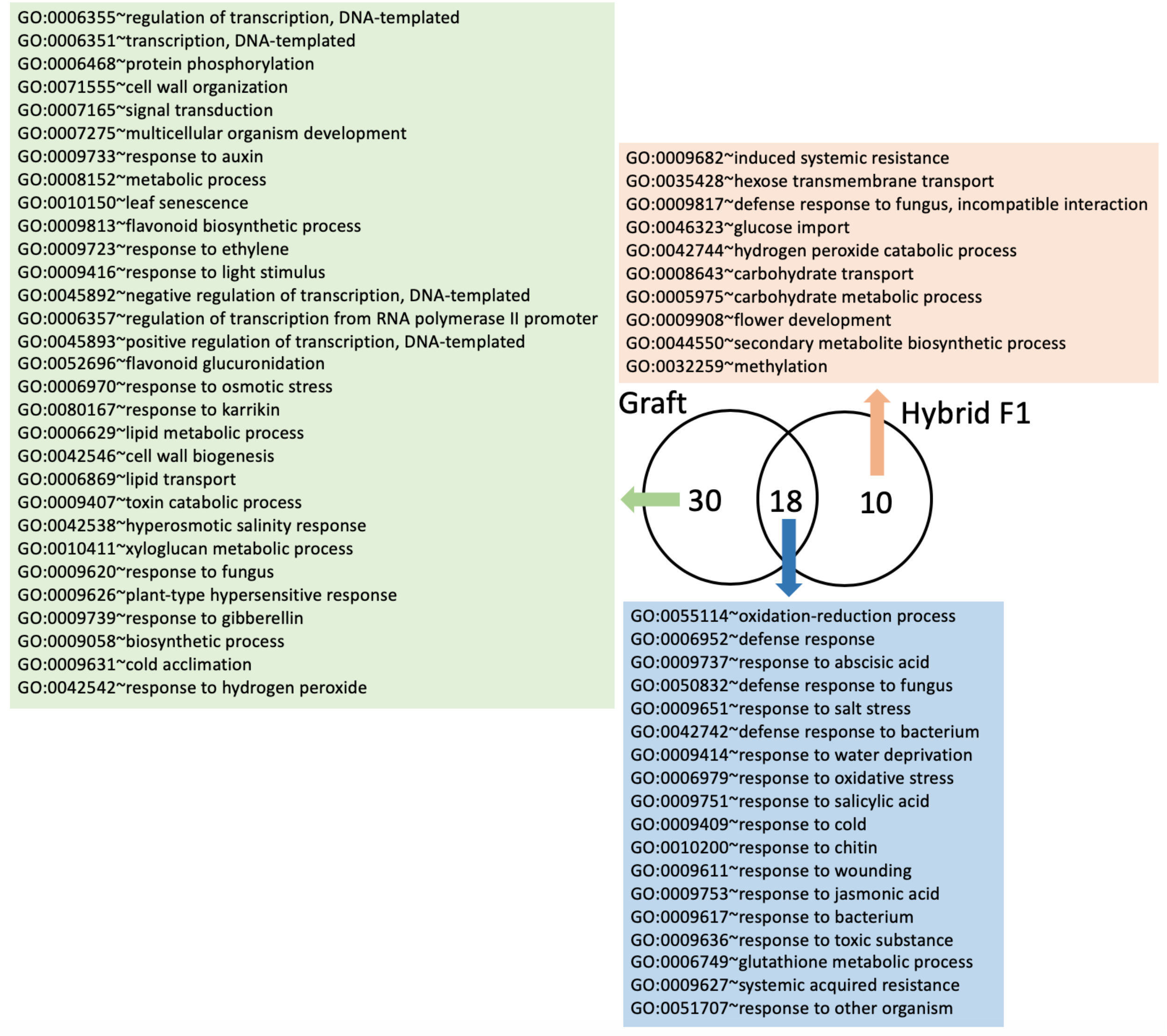

Supplementary Fig. 6. Comparison of enriched GO pathways for DEGs from the HEG effect and Ler × C24 F1 hybrids. The venn diagram of enriched GO pathways from the Arabidopsis graft progeny (first generation) Col-0/Col-0 vs Col-0/*msh1* DEG dataset compared to enriched GO pathways from the Ler × C24 F1 hybrid vs the MPV (Middle parent value) DEG dataset[19]. GO biological process enrichment categories above the cutoff, FDR<0.01, are shown. DAVID GO was used to conduct the analysis.

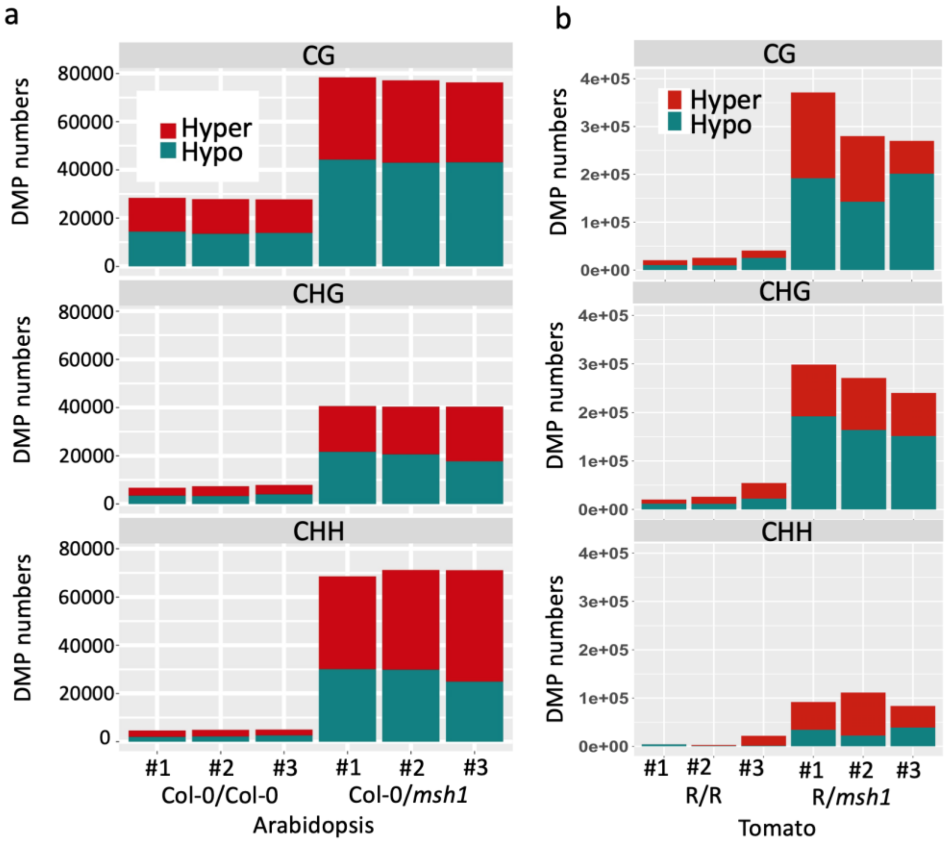

Supplementary Fig. 7. DMP counts in Arabidopsis and tomato graft progenies. a, Total hyper- and hypomethylation DMP counts of first-generation grafted progenies in the Col-0/Col-0 vs Col-0/*msh1* comparison. DMPs were classified as hyper if the site methylation difference in comparisons of each individual to the average of reference plants (the centroid of the Col-0/Col-0 grafted progenies) was greater than 0 and defined as hypo if less than 0. **b**, Total hyper- and hypomethylation DMP counts for first-generation grafted progeny in the Rutgers/Rutgers vs Rutgers/*MSH1*-RNAi comparison. Each bar graph represents a single plant. CG, CHG, CHH context shown separately for each plant. DMPs were defined as hyper if the site methylation difference in comparisons of each individual to the average of reference plants (the centroid of the Rutgers/Rutgers grafted progenies) was greater than 0 and defined as hypo if less than 0. Source data are provided as a Source Data file.

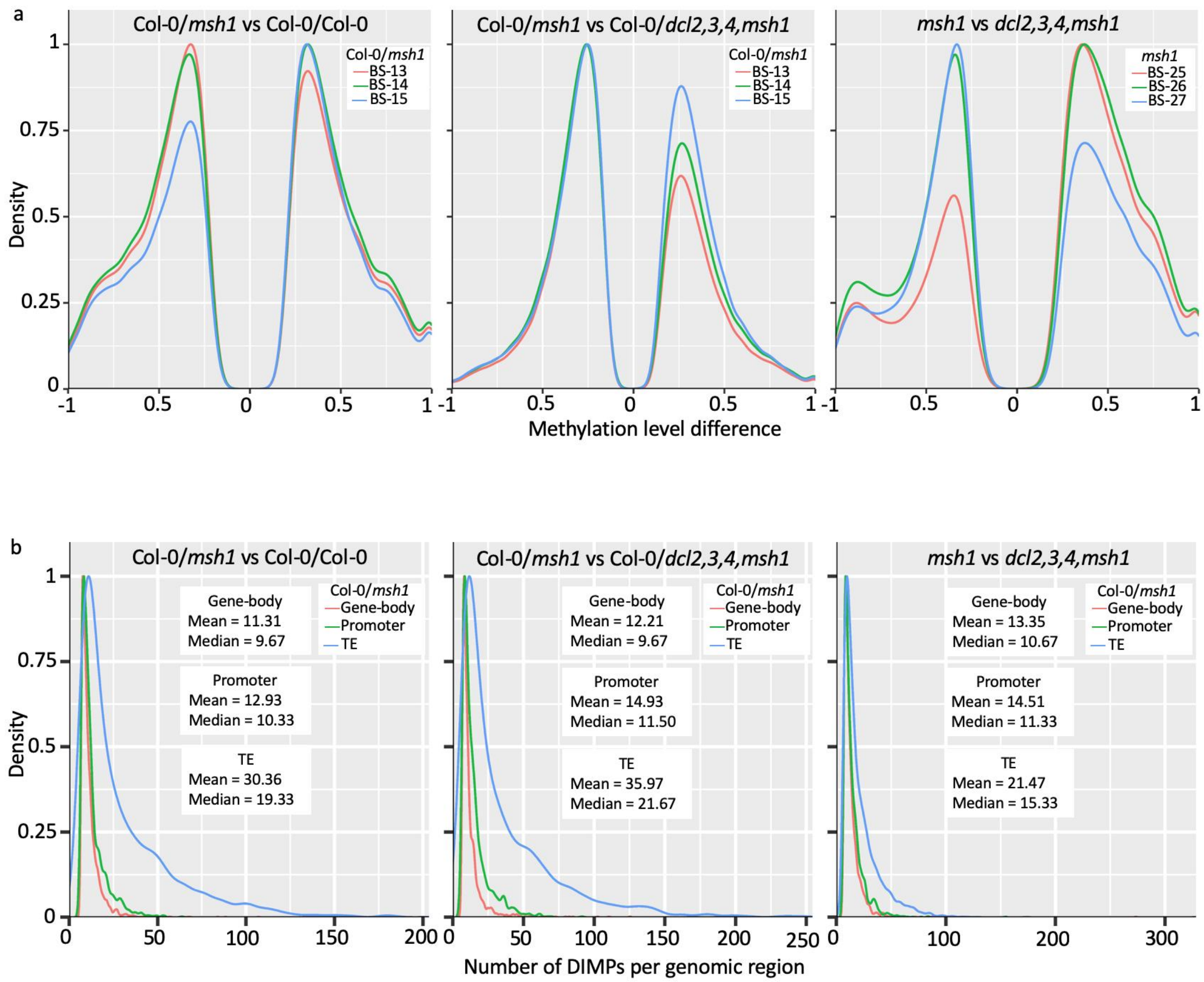

Supplementary Fig. 8. Density plot of DMP methylation level difference and DMP number in DMGs and DMTEs. a, Density plot of DMP methylation level difference. Methylation level differences at each cytosine were computed by subtracting average methylation level of reference plants from the methylation level of each individual sample. The minimum methylation level difference is 0.2. **b**, Density plot of DMP number in DMGs. The average DMP number in DMGs (gene-body and promoter) and DMTEs. For both a and b, scaled density plots using kernel density estimation algorithm were generated by the geom_density function from ggplot2 (R package). Sample size n=3 biological replicates.

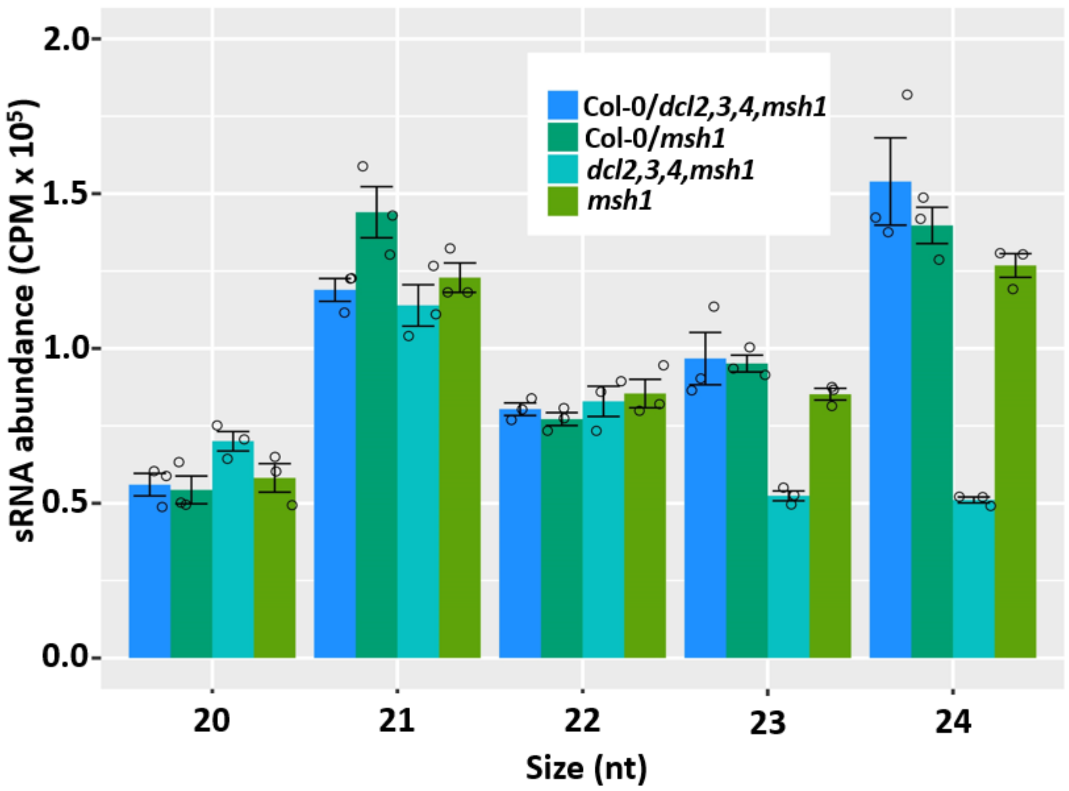

Supplementary Fig. 9. sRNA abundance in grafts and rootstocks. Small RNA abundance (RPM) in *msh1* and *dcl2,3,4,msh1* quadruple mutant rootstock and Col-0/*msh1* and Col-0/*dcl2,3,4,msh1* graft progenies. Using ShortStack, reads were aligned and resulting bam files were used to count uniquely mapped reads for each 20-24nt size sRNA. Read counts were normalized based on the library size using edgeR (R package). Circle on the bar graph represents a sample. Bars represent means ± SE, from 3 biological replicates. Source data are provided as a Source Data file.

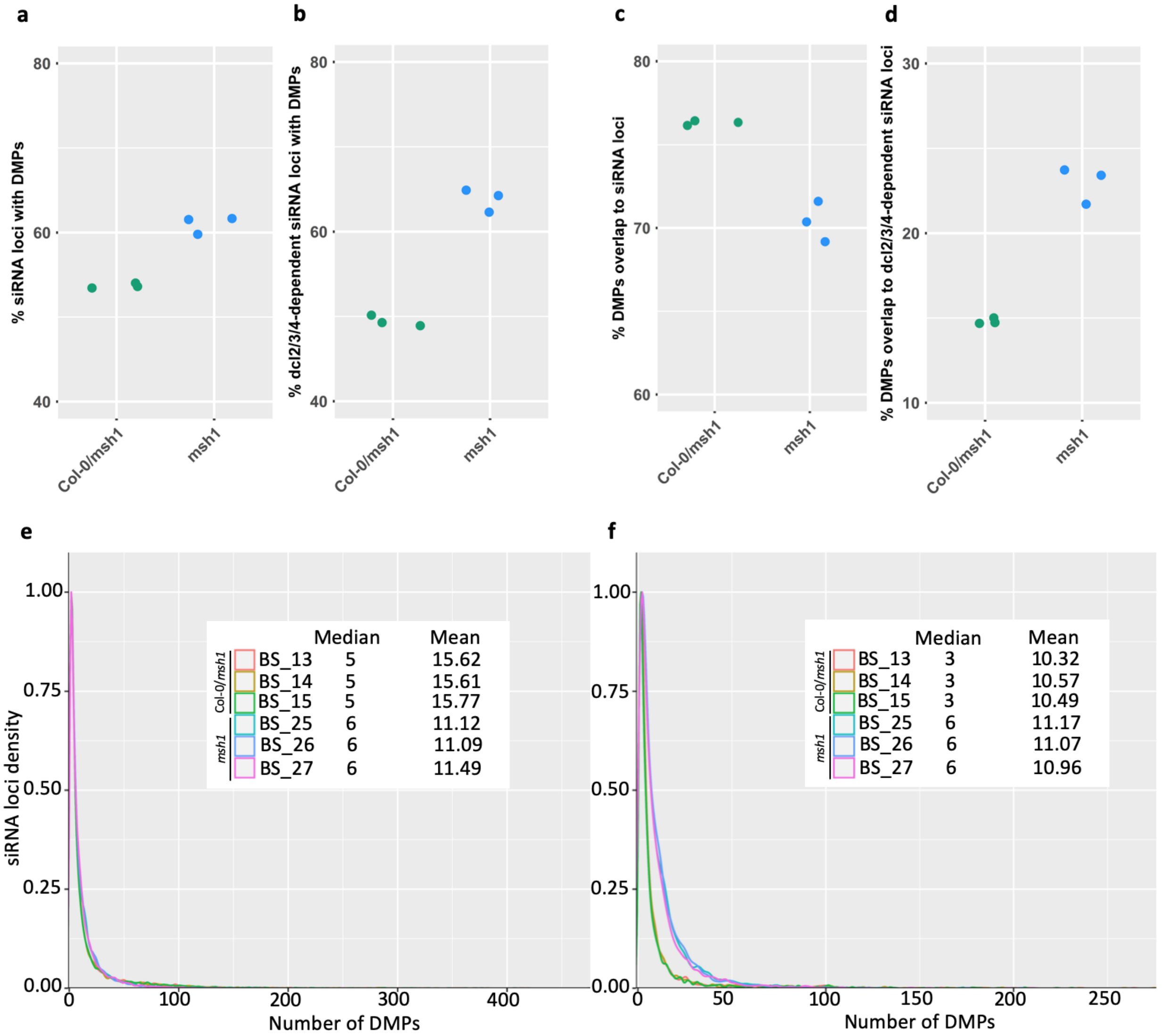

Supplementary Fig. 10. Estimate of siRNA loci and DMP overlap in graft and rootstock. a, Percentage of total siRNA loci with at least one or more differentially methylated position (DMP). **b**, Percentage of *dcl2/3/4*-dependent siRNA loci with at least one or more DMP. **c**, Percentage of total DMPs residing within the total population of siRNA loci. **d**, Percentage of total DMPs residing within the *dcl2/3/4*-dependent siRNA loci. The total population of siRNA loci was identified by ShortStack (version 3.8.3); *dcl2/3/4*-dependent siRNA loci were identified by differential expression analysis between *msh1* and *dcl2,3,4,msh1*. DMPs in Col-0/*msh1* and *msh1* were identified through comparison with Col-0/*dcl2,3,4,msh1* and *dcl2,3,4,msh1*, respectively (Fig. 4e).**e**, Density plot of the total population of siRNA loci and DMPs in Col-0/*msh1* graft (BS_13, BS_14, BS_15) and *msh1* rootstock (BS_25, BS_26, BS_27). **f**, Density plot of *dcl/*2/3/4-dependent siRNA loci and DMPs in Col-0/*msh1* graft (BS_13, BS_14, BS_15) and *msh1* rootstock (BS_25, BS_26, BS_27). Total population of siRNA loci was identified with ShortStack (version 3.8.3); *dcl2/3/4*-dependent siRNA loci were identified by analysis of differential expression between *msh1* and *dcl2,3,4,msh1*. DMPs in Col-0/*msh1* and *msh1* were identified through comparison with Col-0/*dcl2,3,4,msh1* and *dcl2,3,4,msh1*, respectively (Fig. 4e).

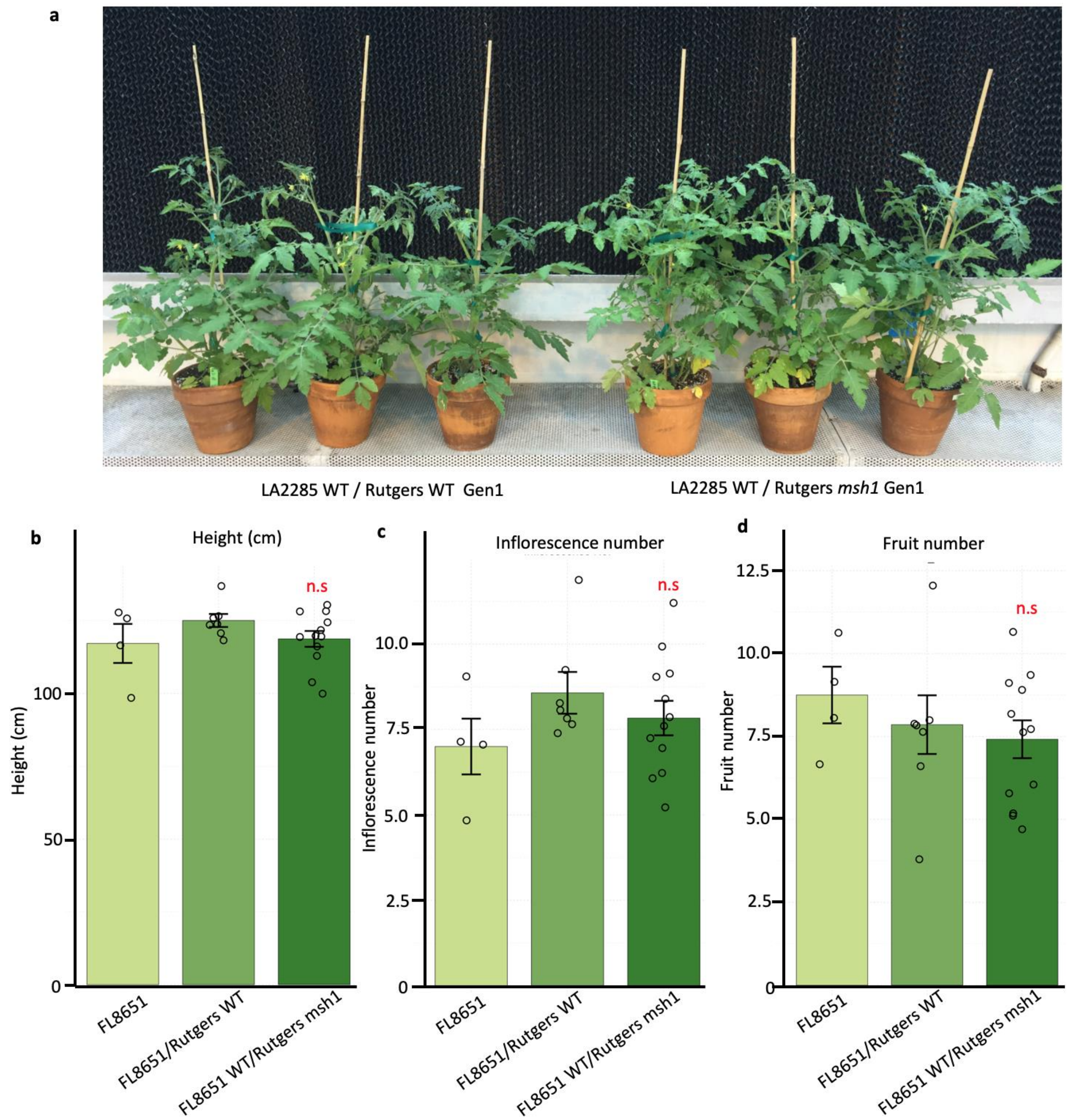

Supplementary Fig. 11. Inter-cultivar grafting experiments in tomato.
a, Phenotype of tomato inter-cultivar graft progenies (first generation) from control graft LA2285 WT / Rutgers WT, and LA2285 WT / Rutgers *msh1,* where wild type plants of South American cultivar LA2285 was used as scion and Rutgers wild type and MSH1-RNAi as contrasting rootstocks. Photo shows plants at 5 weeks. **b-d**, Phenotype of Florida elite cultivar Fla.8651, and inter-cultivar graft progenies (first generation) from control graft Fla.8651WT / Rutgers WT and Fla.8651 WT / Rutgers *msh1,* with wild type Fla.8651 as scion and Rutgers wild type and MSH1-RNAi rootstocks. **b**, Plant height of 5-week- old plants, **c,** inflorescence number of 10-week-old plants and **d**, fruit number of 14-week-old plants. Error bar represents standard error between individual plant replicates (n > 3). No significant difference was found in the mean of Fla.8651/ Rutgers *msh1* vs Fla.8651/ Rutgers WT with a two tailed Student's t-test. Figure shows a representative test of graft progenies from 28 independent graft events with LA0134C, LA1162, LA2285, and five elite cultivars, Fla.8872, Fla.8917, Fla.8651, Fla.7804, Fla.8059, as scion on Rutgers *MSH1*-RNAi rootstock. Source data underlying Supp.Fig.11 b-d are provided as a Source Data file.

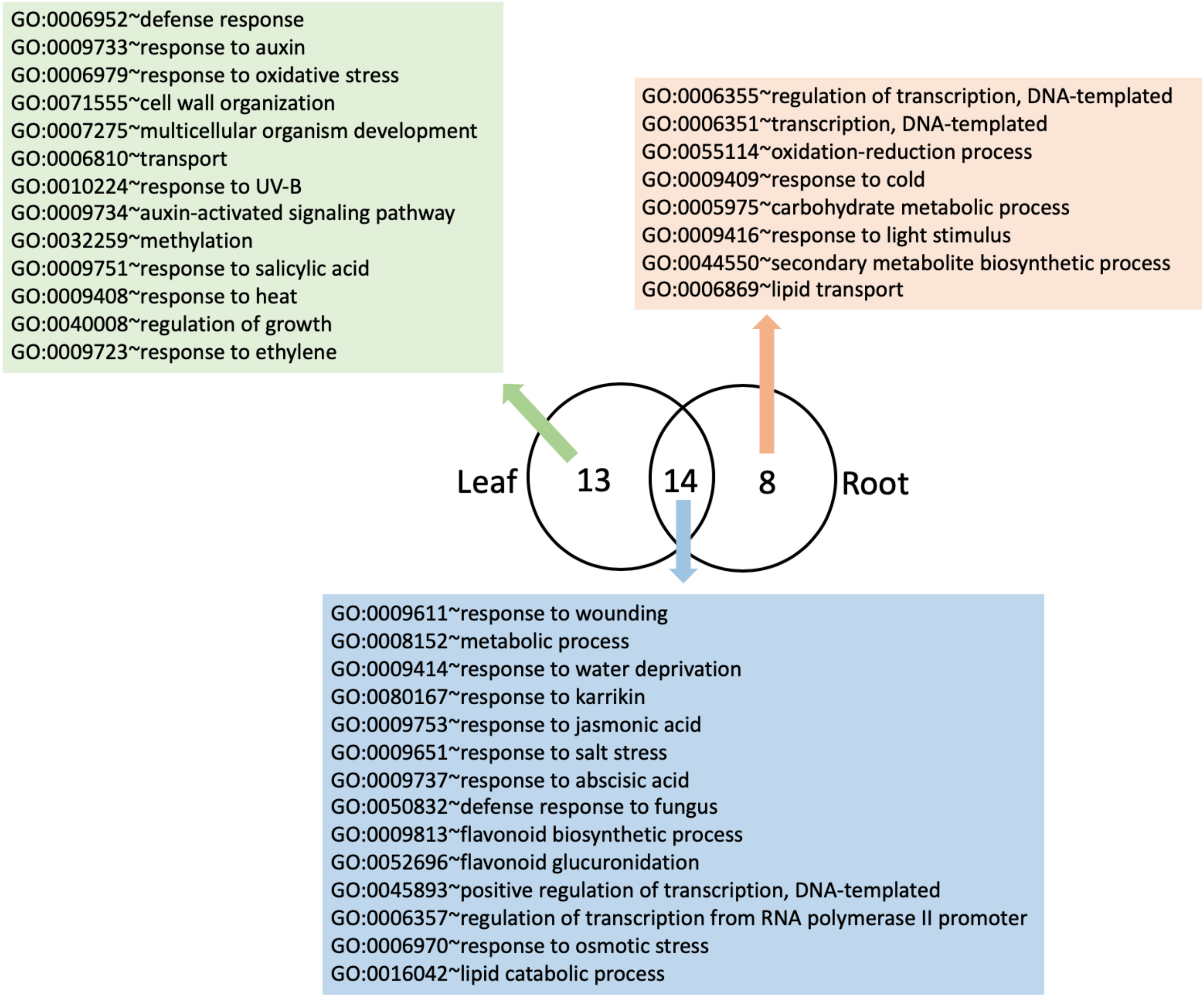

Supplementary Fig. 12. Identified significant enriched GO pathways. Venn diagram shows enriched GO pathways from differentially expressed gene datasets for above-ground leaf tissue compared to DEG datasets for root tissue in the Arabidopsis (first generation) Col-0/*msh1* vs Col-0/*dcl2,3,4,msh1* graft progeny comparison. GO biological process enrichment categories above the cutoff, FDR<0.01, are shown. DAVID GO was used to conduct the analysis.

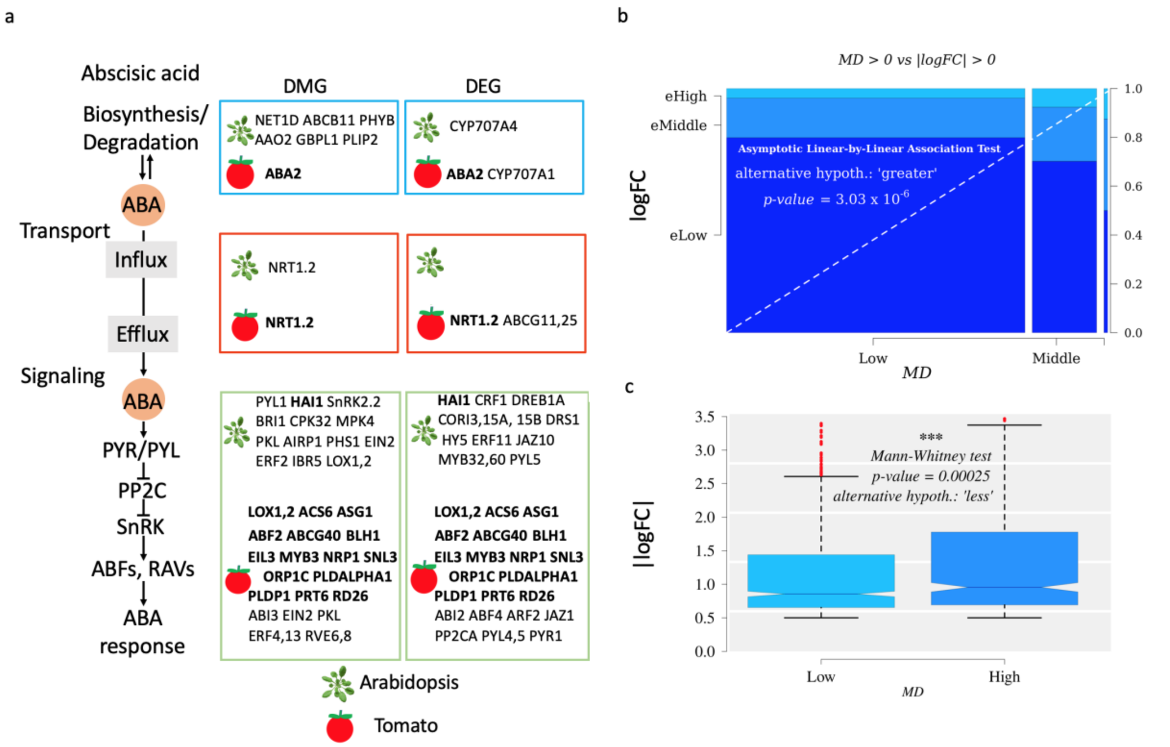

Supplementary Fig. 13. Association of gene expression changes with methylation variation. a, Abscisic acid pathway genes identified in association with the HEG effect. ABA pathway differentially methylated genes (DMGs) and differentially expressed genes (DEGs) were identified in the Arabidopsis Col-0/*msh1* vs Col-0/*dcl2,3,4,msh1* graft progeny (first generation) comparison and tomato R/R vs R/*msh1* graft progeny (first generation) comparison. Genes within the blue box are ABA biosynthesis/degradation related, in the red box are transport-related, and in the green box are signaling-related. Genes identified as both DEGs and DMGs are in bold. **b**, Association between gene expression and the density of methylation for tomato. Spine plot and Linear-by-Linear Association (*lbl*) test applied to evaluate the association between gene expression (logarithm of fold change, *logFC*) and the difference in density of methylation levels on genes (*MD*). A spine plot qualitatively visualizes the linear associations; the rectangular area of each tile is proportional to the frequency of genes at the corresponding category level. A dashed white line highlights the linear trend in the association between *logFC* and *MD*. The *lbl* tests evaluate whether the observed linear trend is statistically significant. Both variables, *logFC* and *MD* were discretized to three levels: *Low*, *Middle*, and *High* (with a *K*-means algorithm), and a one-tail test was carried out (alternative hypothesis: "less"). **c**, A one-sided Mann-Whitney test applied to the dataset from **b**. Data are represented as boxplots where the middle line is the median, the lower and upper hinges correspond to the first and third quartiles, the upper whisker extends from the hinge to the largest value and the lower whisker extends from the hinge to the smallest value, while data beyond the end of the whiskers are outlying points that are plotted individually; sample size n=3 biological replicates. The *logFC* was split to two levels: *Low* and *High MD* values (*Middle* and *High* categories combined). A statistically significant linear trend indicated by the *lbl* test is consistent with a significant shift (lower than zero) of the *logFC* distribution in the *Low MD* gene set relative to *High MD* gene set. Distributions of *logFC* at low and at high *MD* values differ by a location shift μ, with statistically significant differences detected between the distributions of *logFC* values from the two *MD* levels. Supplementary Fig.13a was made by authors based on data from this study and information adopted from Zubo and Schaller[77].

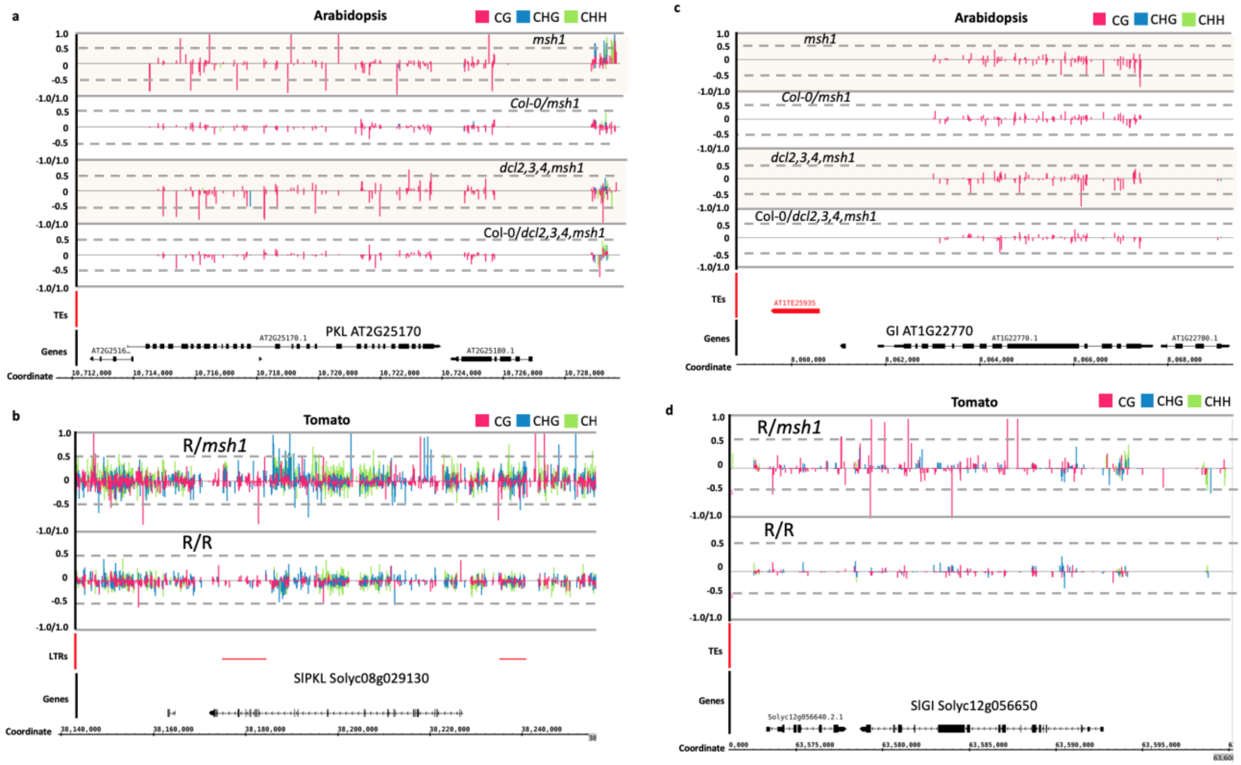

Supplementary Fig. 14. Methylation changes at sample DMG loci. Single cytosine methylation level changes in Arabidopsis *msh1* mutant, *dcl2,3,4,msh1* mutant, and Col-0/*msh1* and Col-0/*dcl2,3,4,msh1* graft progeny (first generation) at (**a**) *PKL* (AT2G25170) and (**c**)*GI* (AT1G22770) loci. Single cytosine methylation level changes in the tomato Rutgers/ Rutgers (R/R) and Rutgers/*MSH1*-RNAi(R/*msh1*) graft progeny (first generation) at the (**b**), *SlPKL*(Solyc08g029130) and (**d**) *SlGI*(Solyc12g056650) loci. Methylation level difference at each cytosine is computed by subtracting average methylation level of reference plants from the methylation level of each individual sample. For *msh1* and *dcl2,3,4,msh1* mutants, *dcl2,3,4,msh1* plants were used as reference. For Col-0/*msh1* and Col-0/*dcl2,3,4,msh1* graft progeny, Col-0/*dcl2,3,4,msh1* graft progeny plants were used as the reference. Only one plant from each genotype was selected as representative; the pattern will differ slightly for different individuals due to fluctuation in methylation. Integrated Genome Browser (version 9.0.2) was used to generate figure.
