# Supplementary material for: MSH1-induced heritable enhanced growth vigor through grafting is associated with the RdDM pathway in plants
Source: Nat Commun. 2020 Oct 22;11:5343. doi: 10.1038/s41467-020-19140-x (PMC7582163; doi:10.1038/s41467-020-19140-x)
Supplement: Supplementary file 3 — Reporting Summary [file 41467_2020_19140_MOESM3_ESM.pdf]

nature research

Corresponding author(s): Sally Mackenzie

Last updated by author(s): Sep 20, 2020

Reporting Summary

Nature Research wishes to improve the reproducibility of the work that we publish. This form provides structure for consistency and transparency in reporting. For further information on Nature Research policies, see our Editorial Policies and the Editorial Policy Checklist.

Statistics

For all statistical analyses, confirm that the following items are present in the figure legend, table legend, main text, or Methods section.

n/a	Confirmed	
☐	☒	The exact sample size (n) for each experimental group/condition, given as a discrete number and unit of measurement
☐	☒	A statement on whether measurements were taken from distinct samples or whether the same sample was measured repeatedly
☐	☒	The statistical test(s) used AND whether they are one- or two-sided<br>*Only common tests should be described solely by name; describe more complex techniques in the Methods section.*
☐	☒	A description of all covariates tested
☐	☒	A description of any assumptions or corrections, such as tests of normality and adjustment for multiple comparisons
☐	☒	A full description of the statistical parameters including central tendency (e.g. means) or other basic estimates (e.g. regression coefficient) AND variation (e.g. standard deviation) or associated estimates of uncertainty (e.g. confidence intervals)
☐	☒	For null hypothesis testing, the test statistic (e.g. F, t, r) with confidence intervals, effect sizes, degrees of freedom and P value noted<br>*Give P values as exact values whenever suitable.*
☒	☐	For Bayesian analysis, information on the choice of priors and Markov chain Monte Carlo settings
☐	☒	For hierarchical and complex designs, identification of the appropriate level for tests and full reporting of outcomes
☒	☐	Estimates of effect sizes (e.g. Cohen's d, Pearson's r), indicating how they were calculated

Our web collection on statistics for biologists contains articles on many of the points above.

Software and code

Policy information about availability of computer code

Data collection	No software are used in the data collection
Data analysis	Numerous commercial programs were used and listed in Methods: SAMtools(version1.9), edgeR(version 3.26.8), Integrated Genome Browser (version 9.0.2), RNA alignment (STAR (version 2.7.3a), sRNA analysis (ShortStack version 3.8.3), RNA read count data (QoRTs software package (version v1.3.0)), Bisulfite sequence used FastQC (version 0.11.5), trimmed with TrimGalore! (version 0.4.1) and Cutadapt (version 1.15), aligned with Bismark (version 0.19.0) with bowtie2 (version 2.3.3.1).<br>For network based enrichment, NEAT (R package version 1.1.3) was used.<br>To identify the homologs of tomato genes in Arabidopsis BLASTP (version 2.9.0) was used. Custom wrapper of BLASTP used for this study is available at https://github.com/genomaths/genomaths.github.io/tree/master/blastp.<br>Statistical analysis for the tomato field data involving linear mixed model was implemented using 'lmerTest' R package (version 3.1-2).<br>ImageJ (https://imagej.nih.gov/ij/index.html) was used to calculate Arabidopsis leaf area.<br>Custom Methyl-IT platform (R package Methyl-IT (version 0.3.2) at https://github.com/genomaths/MethylIT.

For manuscripts utilizing custom algorithms or software that are central to the research but not yet described in published literature, software must be made available to editors and reviewers. We strongly encourage code deposition in a community repository (e.g. GitHub). See the Nature Research guidelines for submitting code & software for further information.

April 2020

Data

Policy information about availability of data

All manuscripts must include a data availability statement. This statement should provide the following information, where applicable:

- Accession codes, unique identifiers, or web links for publicly available datasets
- A list of figures that have associated raw data
- A description of any restrictions on data availability

Data supporting the findings of this work are available within the paper and its Supplementary Infor-mation files. A reporting summary for this Article is available as a Supplementary Information file. The datasets generated and analyzed during the current study are available from the corresponding author upon request. All next-generation sequencing data generated by this study were deposited to Gene Ex-pression Omnibus database with the primary accession code GSE152570 (https://www.ncbi.nlm.nih.gov/geo/query/acc.cgi?acc=GSE152570). Arabidopsis and Tomato genome used as reference are available at http://ensemblgenomes.org/. The source data used for Figs. 1c-e, 1g-i, 2a, 3, 4b-f, 5, and 6b, d as well as Supplementary Figs. 2-5, 7, 9 are provided as a Source Data file.

Field-specific reporting

Please select the one below that is the best fit for your research. If you are not sure, read the appropriate sections before making your selection.

- [x] Life sciences
- [ ] Behavioural & social sciences
- [ ] Ecological, evolutionary & environmental sciences

For a reference copy of the document with all sections, see nature.com/documents/nr-reporting-summary-flat.pdf

Life sciences study design

All studies must disclose on these points even when the disclosure is negative.

Sample size	To assess the phenotypes (leaf area, flowering, and seed yield) of graft progenies, 12-18 plants in each graft population were used for Arabidopsis similar to earlier published study by Virdi et. al., 2015.<br>For tomato field trials, 3-15 plot replicates for each graft population with 12 plants in each plot were used to measure traits. Field trials were carried out in 3 different locations. Tha sample size was selected based on the earlier study by Yang et. al., 2015.<br>For TIBA treatment, Arabidopsis population size was 7-11 plants in each graft population; for tomato population size was 4-9 plants.<br>For BS-seq, RNA-seq, and sRNA-seq, 3 plants were sequenced from each graft combination as it is standard in the research community and is similar to earlier study by Virdi et. al., 2015.
Data exclusions	No data were excluded.
Replication	Grafting experiment was successfully replicated independently from the previously published studies in Virdi et. al., 2015 for Arabidopsis and Yang et. al., 2015 for tomato. For each graft combination at least 4 independent grafts were generated.<br>All the sequencing data were generated from 3 biological replicates.
Randomization	Tomato field trials were carried out in a randomized complete block design (RCBD) in a commercial filed setup.<br>Arabidopsis plants were randomized in a tray along with controls. For better environmental control plants were grown in a reach-in chamber.
Blinding	Lab scientists worked with plant identity and phenotype data. Identity of plants was not revealed until the final data collection as it was required to take pictures for publication in a more organized way. Computational biologists worked blind with coded data.

Reporting for specific materials, systems and methods

We require information from authors about some types of materials, experimental systems and methods used in many studies. Here, indicate whether each material, system or method listed is relevant to your study. If you are not sure if a list item applies to your research, read the appropriate section before selecting a response.

Materials & experimental systems

n/a	Involved in the study	
x		Antibodies
x		Eukaryotic cell lines
x		Palaeontology and archaeology
x		Animals and other organisms
x		Human research participants
x		Clinical data
x		Dual use research of concern

Methods

n/a	Involved in the study	
x		ChIP-seq
x		Flow cytometry
x		MRI-based neuroimaging
