# Supplementary material for: MSH1-induced heritable enhanced growth vigor through grafting is associated with the RdDM pathway in plants
Source: Nat Commun. 2020 Oct 22;11:5343. doi: 10.1038/s41467-020-19140-x (PMC7582163; doi:10.1038/s41467-020-19140-x)
Supplement: Supplementary file 4 — Description of Additional Supplementary Files [file 41467_2020_19140_MOESM4_ESM.pdf]

**Description of Additional Supplementary Files**

File name: Supplementary Data 1

Arabidopsis graft progeny Col0-msh1 versus Col0-Col0 DEGs from leaf tissues

File name: Supplementary Data 2

Tomato graft progeny R/msh1 versus R/R leaf tissue DEGs and unique Arabidopsis homologs

File name: Supplementary Data 3

Arabidopsis grafting progeny Col-0 on msh1 vs Col-0 on Col-0 DMGs from leaf tissue.

File name: Supplementary Data 4

Tomato graft progeny R/msh1 versus R/R 2681 DMGs and their unique Arabidopsis homologs

File name: Supplementary Data 5

Arabidopsis mutant msh1 vs dcl1,2,4,msh1 DMGs

File name: Supplementary Data 6

Arabidopsis graft progeny Col0/msh1 versus Col0/dcl2,3,4,msh1 DMGs

File name: Supplementary Data 7

Arabidopsis mutant msh1 vs dcl2,3,4,msh1 diff. expressed sRNA clusters

File name: Supplementary Data 8

Arabidopsis graft progeny Col0/msh1 versus Col0/dcl2,3,4,msh1 differentially expressed sRNA clusters

File name: Supplementary Data 9

siRNA clusters overlapping graft progeny gene-body DMGs

File name: Supplementary Data 10

Arabidopsis mutant msh1 vs dcl234 msh1 DMTE

File name: Supplementary Data 11

Arabidopsis graft progeny Col0-msh1 vs Col0-dcl2,3,4msh1 DMTEs

File name: Supplementary Data 12

Arabidopsis graft progeny Col0-msh1 versus Col0-dcl2,3,4,msh1 DEGs from root tissues
