# Supplementary material for: MSH1-induced heritable enhanced growth vigor through grafting is associated with the RdDM pathway in plants
Source: Nat Commun. 2020 Oct 22;11:5343. doi: 10.1038/s41467-020-19140-x (PMC7582163; doi:10.1038/s41467-020-19140-x)
Supplement: Supplementary file 2 — Peer Review File [file 41467_2020_19140_MOESM2_ESM.pdf]

Reviewer #1 (Remarks to the Author):

In this manuscript Kundariya et al investigated the molecular basis of the heritable enhancement-through-grafting (HEG) phenomenon using the MSH1 system in Arabidopsis and tomato. This study involved multi-generation, multi-year, and multi-location field trials as well as gene expression, small RNA and whole genome DNA methylation analyses. The authors found that msh1 grafting can induce heritable enhanced growth vigor that is dependent on the RNA-directed DNA methylation (RdDM) pathway. Overall, the experiments were well designed, and the conclusions seem reasonable.

Major concerns:
The title is misleading because the msh1 genetic background was used to study the effect of the RdDM pathway on heritable enhanced growth vigor. This does not warrant for generalization.
Regarding the molecular work, conclusions were drawn mostly from correlations and associations, which were not backed up by additional experiments except for the auxin effect (Fig 6).
The authors claim that genetic relatedness between scion and rootstock may be necessary for enhanced vigor in the graft progeny (page 8, last paragraph, Supplementary figure 10). However, they do not provide any data about the genetic distance between the used tomato genotypes/cultivars.

Additional comments:
For how many generations does the HEG phenomenon last? Can it be boosted by repeated grafting onto the msh1 rootstock?
To aid the readers, the result section should be split into bite sized sections with subheadings.

Reviewer #2 (Remarks to the Author):

This is a very interesting paper. The authors use grafting to show that epigenetic changes in a rootstock from a msh mutant that shows increased vigour are graft transmissible and can be transmitted to the scion and inherited to progeny. Previously the authors published papers describing the msh mutant, graft transmissibility and limited heritability studies. In this paper more extensive heritability studies including multigenerational field trials in a number of locations and the role of epigenetic processes are described. The paper shows graft transmission depends upon the RdDM pathway. The inheritance of this vigour phenotype , particularly the continuous growth vigour over several generations of WT/msh1 graft-derived tomato progeny, is convincing (although it is not clear how the tested lines were selected and whether the increased vigour phenotypes occur reliably in all or most of the progeny of the WT/msh1 graft).

The enrichment of auxin response pathways in the DMGs and DEGs, and the sharing of DEGs with the heterotic Ler × C24 F1 hybrid, are both very interesting. The loss of growth vigour in the progeny of the Col/msh1, dcl2,3,4 graft is consistent with the importance of mobile sRNAs and RdDM in the phenomenon. The lack of growth vigour in the progeny of grafts with distantly related tomato varieties also supports the involvement of sRNA involvement in the phenomenon and seems to be consistent with the idea that it is not the genetic distance, but the environmental stress-induced epigenetic changes, that accounts for enhanced vigour. These have provided some potential mechanistic insights.

One weakness, like most paper based on genome-wide analysis, is the lack of determination of links between the vigour phenotype and specific genes. But the finding of shared phytohormone response pathways is good, partly compensating.

Another weakness is the lack of analysis of the correlation between DMGs and differential gene expression – are the differentially methylated regions of genes affecting the expression of the associated genes and is the methylation repressive?

While the evidence implies the involvement of mobile sRNA, sRNA movement from root stock to scion is not studied. It is understandably difficult to do so in this case because of the similar genetic background between the msh1 rootstock and the wild type scion. A graft using a RdDM mutant (e.g. ago4 mutant) as the scion would be helpful – if the progeny of this RdDM mutant/msh1 graft shows diminished growth vigour compared to those of the WT/msh1 grafts, then the result strengthens the involvement of sRNA/RdDM. It is possible that the phenomenon is not due to small RNA movement at all, but rather, it is similar to stress-induced priming that requires the sRNA pathway, and this priming may simply be due to systemic movement of the phytohormones from the msh1 rootstock that can trigger the same sRNA-involved epigenetic changes in the scion as in the msh1 stock. In this scenario the lack of growth vigour by the progeny of graft with distantly related tomato variety could be explained by the lack of a similar phytohormone-induced epigenetic target gene set (such as lack of the specific TE sequence in the genes).

One thing the authors can mention is whether or not the seed number and seed size are different between the WT/msh1 and WT/WT grafts, as increased seed size, due to reduced seed number, could increase growth vigour, especially in the first generation progeny.

Figure 3a (the methylation data) is hard to understand – is the Col/Col data calculated against the mean value of all three Col/Col lines, and if so, is this mean value also used for the calculation of the col/msh1?

More minor points

What is DMT2? Line 308

Lines 267-268 need to know the extent of genomic sequence variation between these various cultivars. Also, how different are the various Florida and S American cultivars respectively ie have they really only got two different comparisons here or are the 8 comparisons?

Line 377 - 19% doesn't seem a huge amount

394 using tomato RNAi system they could also ask how many of these changes are inherited in progeny that do not inherit the RNAi construct (ie are not grafted)

Reviewer #3 (Remarks to the Author):

The manuscript by Kundariya and Yang et al approaches the very interesting aspect of graft-transmissible heritable epigenetic phenomenon. Many groups have searched for such effects without much success. Here, they use the msh1 mutant in the rootstock to trigger effects in the scion that are heritable in both Arabidopsis and tomato. This mobile signal is dcl2,3,4 dependent and causes widespread changes in DNA methylation and growth vigor that is heritable for several generations in tomato. The manuscript is well written and experiments generally well done. However, there are aspects that could be improved:

Major issues:
- Arabidopsis experiments appear well done but don't necessary show meiotic heritability since they were only done for one generation. The gametes and developing seeds were still connected to a mutant msh1 rootstock, so could have gained epigenetic marks from this tissue without a need for transmitting marks during meiosis. These marks could then be passed mitotically as the seed develops. A second generation would be required to demonstrate meiotic heritability. Including some secondary generation data would be extremely helpful.
- The tomato experiments show meiotic heritability but is there a possibility that artificial selection could have caused some of their observed phenotypes? For instance, the grafted progeny were selected for a certain trait in each generation (ie height) while a similar selection was not applied to the controls. It would be helpful to know how selection was done and to explain this in the methods section.
- It appears the tomato and Arabidopsis experiments were based on 2-4 original grafts from which many progeny were obtained. In some experiments, it looks like only one graft was made and analysed (ie Figure 4d). Some of these are very low numbers which make it hard to conclude if these are exceptional events or reproducible. If more plants were grafted, then these data should be included in the supplemental. Experiments should have at least three grafted parent plants. n number should be clearly indicated in the methods and/or figure legends.
- The tomato hetero-grafts support the idea that relatedness is important for growth enhancement, but just how different are Rutgers from these various genotypes (Figure S10)? Is there really sufficient differences in their genomes such that siRNAs no longer match? Data to support their claim would be helpful.

Minor issues:
- Line 149. 7mm of rain caused flooding. Did they mean 7cm or 7 inches? 7mm isn't very much rain in a month.
- 19% of DMGs from dcl2,3,4 overlapped with siRNAs (line 257; Figure S9). Are there expression differences in any of these genes?
- Figure 3A, B. Are p values possible to include?
- Figure 5A and 5B. Indicate what genotypes were used to make these data
- Figure 6A and 6B are not consistent. Col/msh1 has longer roots in 6A, but longer roots in 6B. Was there a labelling mistake?
- Figure 7C,E. Is tomato msh1 methylation data available and can be added?
- Figure 7. Do BIG or ARF7 show expression differences? RT-PCR or RNAseq data would be helpful here and for the loci in Figure S12.
- What is the mobile signal that msh1 is promoting or activating? The siRNA abundance in msh1 mutants looks WT (Figure S8) so abundance of siRNAs is not a clear answer. This would be a useful discussion point
- Line 359 – Arabidopsis and tomato contain vascular cambium. Please rephrase this discussion point

KUNDARIYA ET AL. MANUSCRIPT NCOMMS-20-26444-T
RESPONSE TO REVIEWER COMMENTS

Reviewer #1 (Remarks to the Author):

In this manuscript Kundariya et al investigated the molecular basis of the heritable enhancement-through-grafting (HEG) phenomenon using the MSH1 system in Arabidopsis and tomato. This study involved multi-generation, multi-year, and multi-location field trials as well as gene expression, small RNA and whole genome DNA methylation analyses. The authors found that msh1 grafting can induce heritable enhanced growth vigor that is dependent on the RNA-directed DNA methylation (RdDM) pathway. Overall, the experiments were well designed, and the conclusions seem reasonable.

Major concerns:
The title is misleading because the msh1 genetic background was used to study the effect of the RdDM pathway on heritable enhanced growth vigor. This does not warrant for generalization.

Response: The title has been changed to "MSH1-induced heritable enhanced growth vigor through grafting is associated with the RdDM pathway in plants"

Regarding the molecular work, conclusions were drawn mostly from correlations and associations, which were not backed up by additional experiments except for the auxin effect (Fig 6).

Response: While it is true that the scope of genome-wide analysis does not permit us to follow up on every observation with additional experiments, we suggest that the power of this study lies in the genome-wide comparison of two plant species undergoing *msh1* reprogramming, and the implementation of mutants to permit high resolution confirmation of siRNA effects. We have now added an additional grafting experiment to further test the suggestion that *dcl2/3/4* impedes the RdDM pathway by introducing the *drm2* methyltransferase mutant to scion (See Suppl Fig 2). We consider this experiment further confirmation of siRNA effects through the graft.

The authors claim that genetic relatedness between scion and rootstock may be necessary for enhanced vigor in the graft progeny (page 8, last paragraph,

Supplementary figure 10). However, they do not provide any data about the genetic distance between the used tomato genotypes/cultivars.

Response: The tomato germplasm is quite diverse, as documented for most of the lines that we have included in our study. Therefore, we have added two references to clarify this:

Sim S-C, et al. (2012) High-Density SNP Genotyping of Tomato (*Solanum lycopersicum L.)* Reveals Patterns of Genetic Variation Due to Breeding. PLoS ONE 7(9): e45520.

Using a high-density single nucleotide polymorphism (SNP) array, this study found significant genetic difference between Rutgers, which belongs to the vintage group, and fla7804 and fla8059, which belong to the fresh market group.

Domínguez, M., Dugas, E., Benchouaia, M. *et al.* The impact of transposable elements on tomato diversity. *Nat Commun* 11, 4058 (2020). https://doi.org/10.1038/s41467-020-17874-2

This study analyzed 602 tomato accessions, including wild tomato relatives (Wild), wild tomatoes (*S. pimpinellifolium*, SP), early domesticated tomatoes (*S. lycopersicum cerasiforme*, SLC), and cultivated tomatoes (*S. lycopersicum lycopersicum* vintage and modern, SLL) and identified 6,906 TE insertion polymorphisms (TIPs) that result from the mobilization of 337 distinct TE families.

Additional comments:
For how many generations does the HEG phenomenon last? Can it be boosted by repeated grafting onto the msh1 rootstock?

Response: While we do not yet know how long the HEG phenomenon lasts, we have now added second generation data for Arabidopsis in Supplemental Figure 2a. In tomato, we indicate that we have carried this out for five generations to date (Suppl Fig 3)

To aid the readers, the result section should be split into bite sized sections with subheadings.

Response: We have attempted to subdivide the Results section to the specific points that we wish to make, in accordance with Nature Communications guidelines for format. It was not clear how the reviewer thought this might be improved

Reviewer #2 (Remarks to the Author):

This is a very interesting paper. The authors use grafting to show that epigenetic changes in a rootstock from a msh mutant that shows increased vigour are graft transmissible and can be transmitted to the scion and inherited to progeny. Previously the authors published papers describing the msh mutant, graft transmissibility and limited heritability studies. In this paper more extensive heritability studies including multigenerational field trials in a number of locations and the role of epigenetic processes are described. The paper shows graft transmission depends upon the RdDM pathway. The inheritance of this vigour phenotype , particularly the continuous growth vigour over several generations of WT/msh1 graft-derived tomato progeny, is convincing (although it is not clear how the tested lines were selected and whether the increased vigour phenotypes occur reliably in all or most of the progeny of the WT/msh1 graft).

The enrichment of auxin response pathways in the DMGs and DEGs, and the sharing of DEGs with the heterotic Ler × C24 F1 hybrid, are both very interesting. The loss of growth vigour in the progeny of the Col/msh1, dcl2,3,4 graft is consistent with the importance of mobile sRNAs and RdDM in the phenomenon. The lack of growth vigour in the progeny of grafts with distantly related tomato varieties also supports the involvement of sRNA involvement in the phenomenon and seems to be consistent with the idea that it is not the genetic distance, but the environmental stress-induced epigenetic changes, that accounts for enhanced vigour. These have provided some potential mechanistic insights.

One weakness, like most paper based on genome-wide analysis, is the lack of determination of links between the vigour phenotype and specific genes. But the finding of shared phytohormone response pathways is good, partly compensating.

Response: We present evidence of changes in pathways that we believe underpin the phenotype change observed. These pathways are interlinked by identifiable hubs (we mention TOR as one) that are expected to be vital to the shift in growth vigor. But to link the vigor response to one or a few particular genes does not reflect the nature of the effects we observe. We don't consider this a weakness of genome-wide analysis, but rather its strength in providing a more comprehensive view of the network-based phenotype shift.

Another weakness is the lack of analysis of the correlation between DMGs and differential gene expression – are the differentially methylated regions of genes affecting the expression of the associated genes and is the methylation repressive?

Response: From this comment, we are unsure whether the reviewer considers the information contained within Supplementary Figure 13 to be inadequate to this question. We have shown that there is, indeed, a relationship between gene expression and methylation changes. We also discuss the observation that, in our experience, methylome data offers higher resolution of targeted pathways than can be found in gene expression data alone, and we suggest that this observation is the likely consequence of pooled tissue analysis. Many of the gene pathways that we identify by DMG analysis are known to be extremely transient and spatiotemporal in their expression. Figure 7 similarly provides information on those genes within each pathway that display both gene expression and methylation variation by the methods that we employed. Again, we do not consider this a weakness of our analysis, but rather, an indication of the inherent stochasticity and spatio-temporal complexity of these networks. The point we wish to make is that whereas gene expression leaves one with a relatively shallow understanding of the changes occurring, methylome analysis provides surprising resolution that should, in future studies, allow modeling of epigenomic reprogramming during stress and phenotype transitions.

While the evidence implies the involvement of mobile sRNA, sRNA movement from root stock to scion is not studied. It is understandably difficult to do so in this case because of the similar genetic background between the msh1 rootstock and the wild type scion. A graft using a RdDM mutant (e.g. ago4 mutant) as the scion would be helpful – if the progeny of this RdDM mutant/msh1 graft shows diminished growth vigour compared to those of the WT/msh1 grafts, then the result strengthens the involvement of sRNA/RdDM. It is possible that the phenomenon is not due to small RNA movement at all, but rather, it is similar to stress-induced priming that requires the sRNA pathway, and this priming may simply be due to systemic movement of the phytohormones from the msh1 rootstock that can trigger the same sRNA-involved epigenetic changes in the scion as in the msh1 stock. In this scenario the lack of growth vigour by the progeny of graft with distantly related tomato variety could be explained by the

lack of a similar phytohormone-induced epigenetic target gene set (such as lack of the specific TE sequence in the genes).

Response: This is an interesting comment. In response, we have added data from an additional experiment where we have used the RdDM-related methyltransferase *drm2* mutant within the scion of an msh1 graft (Supplementary figure 2c). In like manner to what the reviewer has suggested, incorporation of a *drm2* scion obviates the progeny growth enhancement. These data suggest that any effect conditioned by *msh1* rootstock (phytohormone or otherwise) must run through the RdDM pathway.

One thing the authors can mention is whether or not the seed number and seed size are different between the WT/msh1 and WT/WT grafts, as increased seed size, due to reduced seed number, could increase growth vigour, especially in the first generation progeny.

Response: We have added a statement within the figure legend for Supplementary Figure 4: "No significant difference was observed among the average 100 seed count weights of R/R (0.16 gm), R/DR (0.13 gm and 0.14 gm from two progeny plants from a single graft) suggesting that increased seedling vigor does no derive from seed quality." Similarly, we have observed no differences in Arabidopsis seed weight.

Figure 3a (the methylation data) is hard to understand – is the Col/Col data calculated against the mean value of all three Col/Col lines, and if so, is this mean value also used for the calculation of the col/msh1?

Response: This is correct. The average of 3 control plants (the centroid of Col-0/Col-0 graft progenies) was used as reference for both Col0/Col0 and Col0/msh1 lines. By doing this, we assay the individual variation within the control population and the difference between the Col0/Col0 and Col-/msh1 population simultaneously.

More minor points

What is DMT2? Line 308

Response: DMT2 refers to DNA METHYLTRANSFERASE 2 (AT4G14140), also called MET2. We have now added the gene ID to the text along with the gene name.

Lines 267-268 need to know the extent of genomic sequence variation between these various cultivars. Also, how different are the various Florida and S American cultivars respectively ie have they really only got two different comparisons here or are the 8 comparisons?

Response: we have now added two references that describe the variation across the germplasm that we have incorporated. These lines were included based on their expected variation.

Line 377 - 19% doesn't seem a huge amount (overlap sRNA clusters with DMGs)

Response: We indicate that the greatest amount of sRNA overlap is with TEs. In fact, approximately 64% of the *dcl2,3,4*-dependent siRNA loci are found to be within 1kb of TEs, allowing us to account for 83% of identified siRNA loci. Many TEs are associated with stress response pathways, so we do not consider the sRNA/TE association coincidental. In addition, the 19% only reflect "direct" effects that are observed linking the rootstock with progeny. We anticipate that a large proportion of "indirect" effects occur during reproductive reprogramming, where sRNA effects are demonstrated by others to be pronounced. We do not assay graft reproductive tissues in this study, but focus on *heritable* transmission effects.

394 using tomato RNAi system they could also ask how many of these changes are inherited in progeny that do not inherit the RNAi construct (ie are not grafted)

Response: We are unclear about what the reviewer is asking here. There is no RNAi construct inheritance in this system. The tomato RNAi construct resides within the rootstock, not the scion from which seed is collected. In our previous study (Yang et al. 2020 Nature Comm), we have shown that msh1 memory can occur in plants that do not inherit the RNAi construct. That memory phenomenon is not relevant here.

Reviewer #3 (Remarks to the Author):

The manuscript by Kundariya and Yang et al approaches the very interesting aspect of graft-transmissible heritable epigenetic phenomenon. Many groups have searched for such effects without much success. Here, they use the msh1 mutant in the rootstock to trigger effects in the scion that are heritable in both Arabidopsis and tomato. This mobile signal is dcl2,3,4 dependent and causes widespread changes in DNA methylation and growth vigor that is heritable for several generations in tomato. The manuscript is well written and experiments generally well done. However, there are aspects that could be improved:

Major issues:

- Arabidopsis experiments appear well done but don't necessary show meiotic heritability since they were only done for one generation. The gametes and developing seeds were still connected to a mutant msh1 rootstock, so could have gained epigenetic marks from this tissue without a need for transmitting marks during meiosis. These marks could then be passed mitotically as the seed develops. A second generation would be required to demonstrate meiotic heritability. Including some secondary generation data would be extremely helpful. (second gen TDNA grafts. Leaf area) (Third generation for tomato for marks)

Response: In response to this concern, we have now added additional evidence in Arabidopsis that the msh1 effect is evident in generation 2 populations as well. These data are shown in Supplementary Figure 2. In combination with evidence in the study that the msh1 effect is evident in up to five tomato generations following grafting, it is reasonable to assume that we are observing meiotically transmissible effects.

- The tomato experiments show meiotic heritability but is there a possibility that artificial selection could have caused some of their observed phenotypes? For instance, the grafted progeny were selected for a certain trait in each generation (ie height) while a similar selection was not applied to the controls. It would be helpful to know how selection was done and to explain this in the methods section.

Response: We have now added text to the Methods section to indicate that no phenotypic selection was imposed on these studies. For each generation, seed from the same genotype (and epitype) and plot were

collected, pooled and used for the next field trial without considering previous trial performance.

- It appears the tomato and Arabidopsis experiments were based on 2-4 original grafts from which many progeny were obtained. In some experiments, it looks like only one graft was made and analysed (ie Figure 4d). Some of these are very low numbers which make it hard to conclude if these are exceptional events or reproducible. If more plants were grafted, then these data should be included in the supplemental. Experiments should have at least three grafted parent plants. n number should be clearly indicated in the methods and/or figure legends.

Response: In Arabidopsis, we performed at least three independent grafts for each genotype combination. Because leaf area was used for classification of the HEG phenotype and sequencing, we have many more independent datasets for leaf area than for other phenotypic measurements. In tomato, 5 independent graft events were carried out with Rutgers scion on Rutgers MSH1-RNAi rootstock, and 28 independent grafts involved 8 genetically different cultivars as scion. We have attempted to clarify this information in the text, and have added a Supplementary Figure 2 with additional Arabidopsis grafting experiments to provide more substantiation to the study.

- The tomato hetero-grafts support the idea that relatedness is important for growth enhancement, but just how different are Rutgers from these various genotypes (Figure S10)? Is there really sufficient differences in their genomes such that siRNAs no longer match? Data to support their claim would be helpful.

Response: As indicated in response to the other reviewers, we have added two additional references that clarify the extent of sequence variation in different tomato types, indicating that Rutgers displays significant (by domesticated tomato standards) genetic distance from the genotypes we selected. We have also provided an important recent reference that clarifies that much of the variation in tomato is associated, and surrounds, TE sites within the genome. Of course, we have only hypothesized sequence divergence to be a likely explanation for the loss of msh1 graft effect in these hetero-graft experiments. It will require far more substantial and detailed analysis of the target sites in each genotype, together with functional analysis, to conclusively demonstrate SNP impact.

Minor issues:

- Line 149. 7mm of rain caused flooding. Did they mean 7cm or 7 inches? 7mm isn't very much rain in a month. (please correct)

Response: thank you for pointing this out. We have now clarified this statement, and presented the corrected precipitation amounts in mm rather than inches.

- 19% of DMGs from dcl2,3,4 overlapped with siRNAs (line 257; Figure S9). Are there expression differences in any of these genes?

Response: Yes, this information on coincidence of DMG and DEG data is presented in Figure 7 and Supplementary Figure 13. These data, however, rely on what is detected in DEG datasets derived from pooled above-ground tissues at bolting stage. To test this more definitively will require the development of cell type-specific transcriptome datasets, representing ongoing investigations that extend beyond the scope of the current study.

- Figure 3A, B. Are p values possible to include?

Response: The purpose of this figure is to provide an overview of DMP distribution over the genomic features at an individual plant level. Thus, it would not be statistically meaningful to conduct a test between an individual control and treatment plant.

- Figure 5A and 5B. Indicate what genotypes were used to make these data

Response: Thank you for pointing out this omission. We have now added the genotype information into the figure and figure legend.

- Figure 6A and 6B are not consistent. Col/msh1 has longer roots in 6A, but longer roots in 6B. Was there a labelling mistake?

Response: There was no labelling mistake but the change in sample order might have been confusing to a reader. So we have rearranged the figure so that the order of samples in Figures 6a and 6b agree.

- Figure 7C,E. Is tomato msh1 methylation data available and can be added?

Response: In this figure we made a labelling mistake that we have now corrected. The upper channel of Fig 7c and e should be R/msh1, and the

lower channel should be R/R. This correction now coincides the figure with the Results text. This correction was also made for Supplementary Figure 14b and d. However, we do not have tomato msh1 (RNAi) methylation data.

- Figure 7. Do BIG or ARF7 show expression differences? RT-PCR or RNAseq data would be helpful here and for the loci in Figure S12.

Response: As shown in Figure 7a, BIG displays both differential methylation and expression in tomato. ARF7 undergoes differential methylation in Arabidopsis and tomato, but we do not detect differential expression in pooled tissue sampling. We assume that this is due to the localized and low level expression of this transcription factor in mature vegetative tissues.

- What is the mobile signal that msh1 is promoting or activating? The siRNA abundance in msh1 mutants looks WT (Figure S8) so abundance of siRNAs is not a clear answer. This would be a useful discussion point

Response: It is important to note that we are assaying first generation graft progeny, not the actual scion of the graft where the siRNA are expected to be active. This figure does not include WT, only the relative levels in the lines indicated. In our previous report, Shao et al. 2017 that is cited, we have documented the marked changes in sRNA in *msh1* relative to WT.

- Line 359 – Arabidopsis and tomato contain vascular cambium. Please rephrase this discussion point

Response: Thank you, we have rewritten this for better clarity.

Reviewer #1 (Remarks to the Author):

The revised manuscript addressed most of my concerns. I am happy with the changes made to the manuscript.

Reviewer #2 (Remarks to the Author):

I consider the authors have made sufficient response to satisfy my and other reviewers' queries. Are references 24 and 26 the same?

Reviewer #3 (Remarks to the Author):

Thank you for these revisions, my comments have been satisfactorily addressed.
